# T cell immune discriminants of HIV reservoir size in a pediatric cohort of perinatally infected individuals

**DOI:** 10.1371/journal.ppat.1009533

**Published:** 2021-04-26

**Authors:** Stefano Rinaldi, Lesley de Armas, Sara Dominguez-Rodríguez, Suresh Pallikkuth, Vinh Dinh, Li Pan, Kathleen Gӓrtner, Rajendra Pahwa, Nicola Cotugno, Pablo Rojo, Eleni Nastouli, Nigel Klein, Caroline Foster, Anita De Rossi, Carlo Giaquinto, Paolo Rossi, Paolo Palma, Savita Pahwa, on behalf of EPIICAL consortium

**Affiliations:** 1 Department of Microbiology and Immunology, Miller School of Medicine, University of Miami, Miami, Florida; 2 Hospital Universitario 12 de Octubre, Madrid, Spain; 3 UCL Great Ormond Street Institute of Child Health, London, United Kingdom; 4 Bambino Gesu Children’s Hospital IRCCS, Rome, Italy; 5 Imperial College Healthcare NHS Trust, London, United Kingdom; 6 Istituto Oncologico Veneto IOV-IRCCS, Padova, Italy; 7 University of Padova, Padova, Italy; Emory University, UNITED STATES

## Abstract

The size of the latent HIV reservoir is associated with the timing of therapeutic interventions and overall health of the immune system. Here, we demonstrate that T cell phenotypic signatures associate with viral reservoir size in a cohort of HIV vertically infected children and young adults under durable viral control, and who initiated anti-retroviral therapy (ART) <2 years old. Flow cytometry was used to measure expression of immune activation (IA), immune checkpoint (ICP) markers, and intracellular cytokine production after stimulation with GAG peptides in CD4 and CD8 T cells from cross-sectional peripheral blood samples. We also evaluated the expression of 96 genes in sort-purified total CD4 and CD8 T cells along with HIV-specific CD4 and CD8 T cells using a multiplexed RT-PCR approach. As a measure of HIV reservoir, total HIV-DNA quantification by real-time PCR was performed. Poisson regression modeling for predicting reservoir size using phenotypic markers revealed a signature that featured frequencies of PD-1+CD4 T cells, TIGIT+CD4 T cells and HIV-specific (CD40L+) CD4 T cells as important predictors and it also shows that time of ART initiation strongly affects their association with HIV-DNA. Further, gene expression analysis showed that the frequencies of PD-1+CD4 T cells associated with a CD4 T cell molecular profile skewed toward an exhausted Th1 profile. Our data provide a link between immune checkpoint molecules and HIV persistence in a pediatric cohort as has been demonstrated in adults. Frequencies of PD-1+ and TIGIT+CD4 T cells along with the frequency of HIV-specific CD4 T cells could be associated with the mechanism of viral persistence and may provide insight into potential targets for therapeutic intervention.

## Author summary

Low HIV reservoir size is associated with positive outcomes of therapeutic approaches and better immune function. Here, we identified a 9-marker T cell immune signature based on phenotypic flow cytometry data that associated with total HIV DNA measurements in a pediatric cohort of 34 perinatally infected participants with sustained viral control. Notably, frequencies of PD-1+ CD4 T cells and TIGIT+ CD4 T cells were positively correlated and HIV-specific (CD40L+) CD4 T cells were negatively correlated with HIV DNA, and were impacted by time of ART initiation. Gene expression analysis by multiplex RT-PCR showed that the frequencies of PD-1+ CD4 T cells associated with an exhausted Th1 molecular profile in CD4 T cells. This signature could inform future therapeutic studies and provide mechanistic insight on HIV persistence in perinatally infected HIV.

## Introduction

The introduction of combination Antiretroviral Therapy (ART) has dramatically improved the survival of HIV infected individuals and reduced HIV transmission. However, there are still 38 million people living with HIV (PWH) worldwide and of those 1.8 million are children under the age of 15, highlighting the need for curative therapies. The greatest obstacle to achieve permanent HIV remission is the latent HIV reservoir [1–3]. A smaller viral reservoir is associated with better outcomes to therapeutic intervention [4] (e.g. analytical treatment interruption) and a healthier immune system [5–6].

Memory CD4 T cells are enriched in cell-associated HIV DNA and are known to harbor replication-competent virus [7–9]. Although virus-infected cells constitute a small proportion of CD4 T cells their contribution to the total body latent reservoir is not known. Investigation of immune factors related to HIV persistence has predominantly centered around the CD4 T cell reservoir [10–11]. In this context, cellular markers of immune activation, exhaustion, and HIV-specific T cell responses have been found to be associated with the size of the HIV reservoir [9,12–15].

Immune activation, traditionally defined in T cells by the co-expression of CD38 and HLA-DR, and systemic inflammation are known to increase during the acute phase of HIV infection [16]. Upon initiation of ART the level of T cell activation declines rapidly but does not reach the pre-infection state [17–18].

Immune checkpoint (ICP) molecules are co-inhibitory receptors upregulated during T cell activation to prevent hyper-immune activation and their role is to dampen the immune response [19]. Overexpression of ICPs is indicative of T cell exhaustion and they are upregulated in certain cancers and chronic viral infections including HIV [20–22]. PD-1 expression on T cells, a well characterized ICP marker, has been shown to positively associate with HIV reservoir measurements, such as integrated and total HIV DNA, 2-LTR circles and cell-associated unspliced HIV RNA, and to contribute to HIV persistence [9,13,19,23].

HIV-specific T cell responses are mounted during the course of infection and constantly adapt to virus escape mutations, but they are not effective at clearing the infection. Within HIV-specific T cell responses, the polyfunctional CD8 T cell response is considered to be a correlate of viral control in untreated HIV infected individuals [14,24,25] while expression of ICP markers is associated with loss of functionality in effector cells [22,26]. In fact, blockade of the PD-1 pathway enhances proliferation of HIV-specific T cells as well as improving cytokine responses [20–22,27–28].

At present, early initiation of ART is the most efficient therapeutic intervention to limit seeding of HIV reservoirs. Infants born to HIV infected mothers offer a unique opportunity for ART initiation within hours of infection making this population prime candidates for testing therapeutic interventions for HIV cure [29–31]. However, the majority of studies have been performed in adults and therefore immune correlates of reservoir size and viral control mechanisms in children are lacking. An immune signature associating with these outcomes could be used to identify therapeutic targets or act as an endpoint for evaluating therapies aimed to achieve HIV remission.

The objective of the current study was to generate a T cell immune signature using a computational approach to evaluate the relationships between immune activation, immune checkpoint markers, HIV-specific T cell responses and viral reservoir measurements in a cohort of long-term ART suppressed perinatally HIV-infected adolescent participants that initiated ART within 2 years of life [31–32]. We identified a 9-marker immune signature that was able to discriminate between perinatally infected young individuals with high and low HIV viral burden. Frequencies of PD-1 and TIGIT expressing CD4 T cells along with frequency of HIV-specific CD4 T cells were among the most important predictors of the HIV reservoir.

## Results

We designed three multi-parameter flow cytometry panels to fully characterize phenotype and function of CD4 and CD8 T cells from a cohort of HIV-infected children and adolescent participants on ART (**Table 1**). Flow cytometry panel 1 was designed to investigate immune activation markers (ICOS, HLA-DR, CD38, CD25, CCR5, and Ki-67). Panel 2 assessed immune checkpoint markers (PD-1, LAG3, TIM3, PDL-1, CTLA4, and TIGIT). All phenotypic markers were analyzed as frequencies of total CD4 and CD8 T cells as well as of individual maturation subsets (**S1 and S2A–S2D Figs**). Panel 3 aimed to quantify the HIV-specific (HIVsp) CD4 and CD8 T cell compartments and evaluate their function by intracellular cytokine production after HIV peptide stimulation. To identify HIVsp T cells, we used the expression of activation induced molecules (AIM) CD40L and CD69 on CD4 and CD8 T cells, respectively. Within the AIM+ cells, we measured production of IL-2, IL-21, IFN-γ and TNF-α for CD4 T cells and IL-2, IFN-γ, TNF-α, Perforin, and Granzyme B along with the degranulation marker CD107a for CD8 T cells (**S2E and S2F Fig**). Cell-associated HIV DNA measured in PBMC from the same samples was used as a measurement of HIV reservoir size [31,33–34]. HIV DNA was measured in purified CD4 T cells in a subset of participants (n = 31) and strongly correlated with the PBMC results from the full cohort used in this study (n = 34) (p-value <0.0001, r-value = +0.66).

**Table 1:** Characteristics of study participants.

# individuals	34
**Median Age at sampling** (Range) Years	12.5 (8.4–16.3)
**Median CD4 Absolute Count** (Range) cells/uL	1523 (617.5–2252)
**Median CD8 Absolute Count** (Range) cells/uL	1596 (636.3–2329)
**Median CD4/CD8 ratio** (Range)	0.9 (0.5–1.8)
**HIV-caDNA (Copies/10**^**6**^ **PBMC)**	50 (12.3–323)

### Identification of the immunological predictors of HIV reservoir size

**Fig 1:**
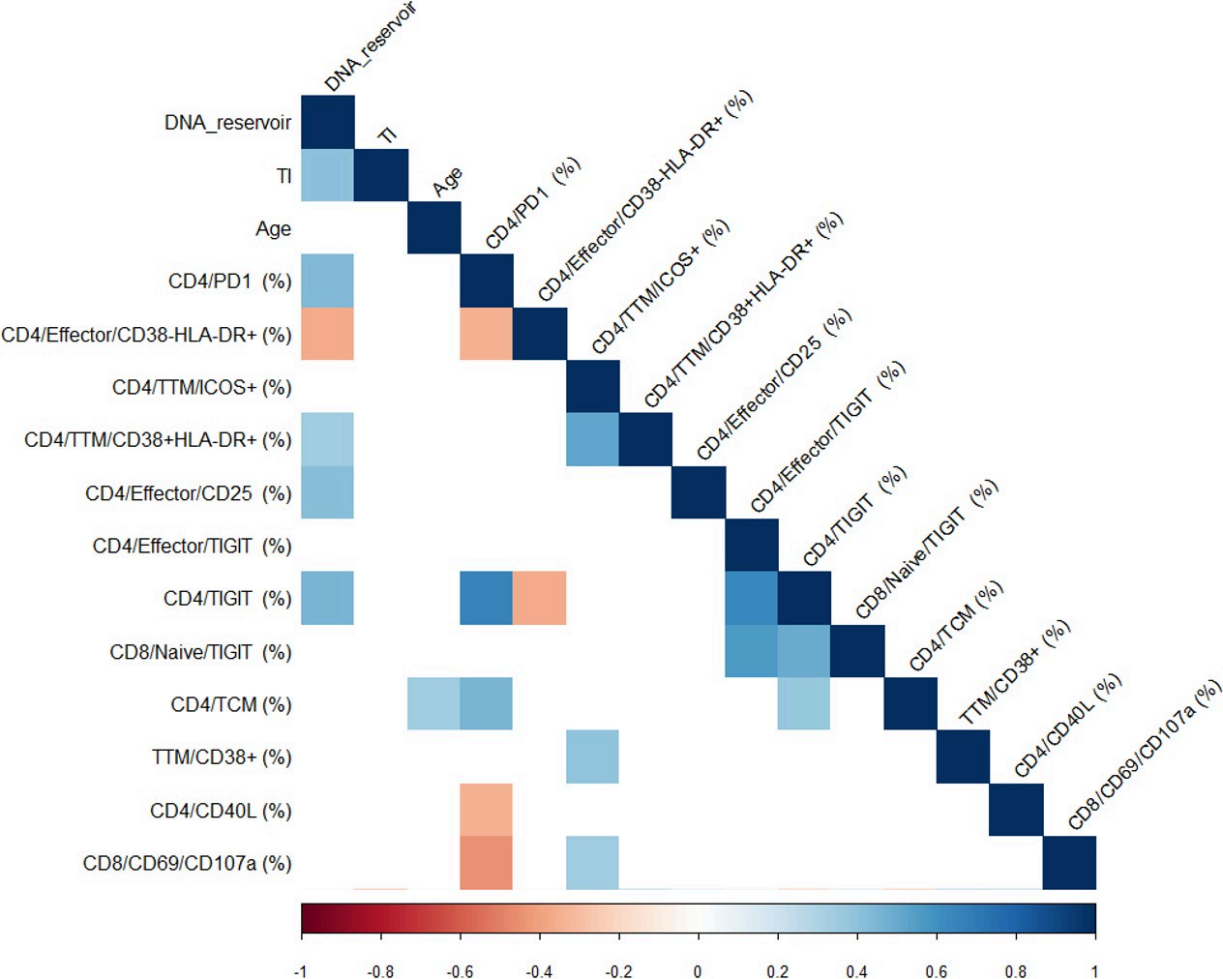
Correlation analysis of Predictors for HIV-1 DNA reservoir size. Correlogram showing the results of the correlation matrix of all the predictors selected by elastic net and DNA reservoir (HIV DNA), TI (Time of treatment initiation) and Age. Spearman r correlation values are shown from red (-1.0) to blue (1.0); r values are indicated by color shade. Blank fields indicate not significant correlations.

Using a penalized regression, variables were selected from the total set of flow cytometry parameters (**S1 Table**) for association with HIV DNA measurement. The selected variables included CD4 Effector/CD38-HLA-DR+, CD4 Transitional Memory (TTM)/ICOS+, TTM/CD38+HLA-DR+, CD4 Effector/CD25+, CD4 Effector/TIGIT+, CD4/TIGIT+, CD8 Naive/TIGIT+, CD4 T central Memory (TCM) and TTM/CD38+ from the *ex vivo* flow cytometry panels, and CD4/CD40L+ and CD8/CD69/CD107a+ from the stimulation flow panel. This approach allows for selection of the best correlates while simultaneously minimizing the effect of co-correlation (**Fig 1**). Next, we used all 11 variables and CD4/PD-1+ to generate a predictive model for HIV reservoir size. CD4/PD-1+ subset was not selected (probably due to co-correlation with other parameters), however we included it *a priori* because of evidence that PD-1 expressing CD4 T cells in literature [6,9,13,23] and in our cohort (**Fig 1**) show strong associations with size of the viral reservoir.

**Fig 2:**
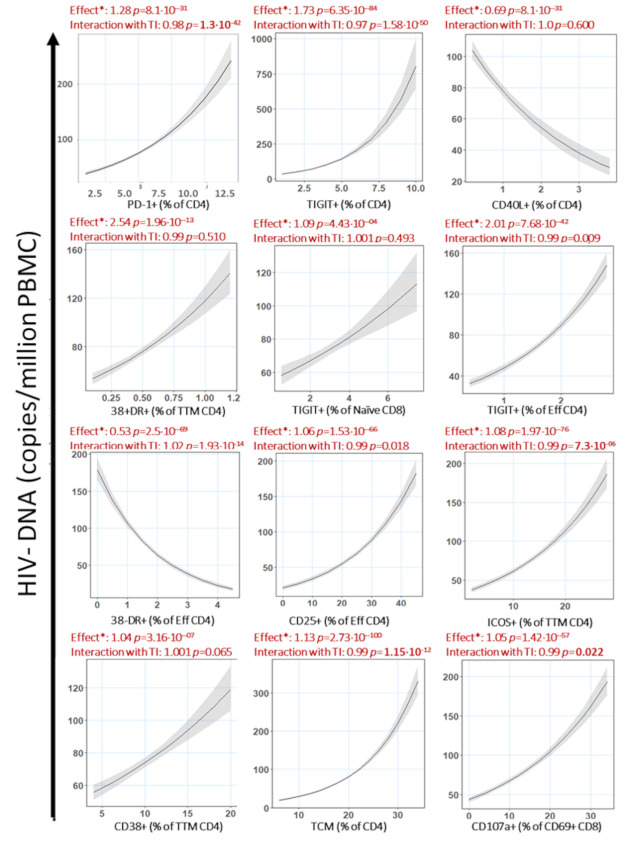
Predictors of HIV-1 DNA reservoir associate with HIV-1 DNA reservoir and interact with age at Treatment initiation (TI). Predictors identified by penalized regression were plotted against the predicted outcome (HIV-DNA) based on the multivariable Poisson regression model after adjusting for baseline CD4, baseline viral load, age at the reservoir measurement and age at ART. *: effect of the association between the parameter and the reservoir. By increasing one unit the predictor a X% increased of the reservoir is predicted. A value of effect above 1 indicates a positive contribution while a value between 0 and 1 indicates a negative contribution. Interaction of immune variable plotted with time of treatment initiation (TI) is also indicated on top.

To describe the individual association between the predictors and size of the HIV DNA reservoir, we applied multivariable Poisson regression adjusting for nadir CD4, baseline plasma viral load, age at the time of reservoir measurement and age at the time of treatment initiation (TI) (**Fig 2)**. The effect determined for each predictor on HIV DNA levels is that, maintaining all the other predictors the same, an increase in the value of an individual predictor of 1 unit will correspond to a proportional increase equal to (effect -1) * 100. Following this rule, an effect above 1 is always equal to an increase in the predicted variable while an effect below 1 is always equal to a decrease in the predicted variable. For example, CD4/ PD-1+ T cells as a predictor has an effect of 1.28 on HIV DNA, which can be interpreted as an increase of 1% in the frequency of PD1+ CD4 T cells is predicted to correspond to a 28% increase in HIV DNA copies/million PBMC.

**Fig 3:**
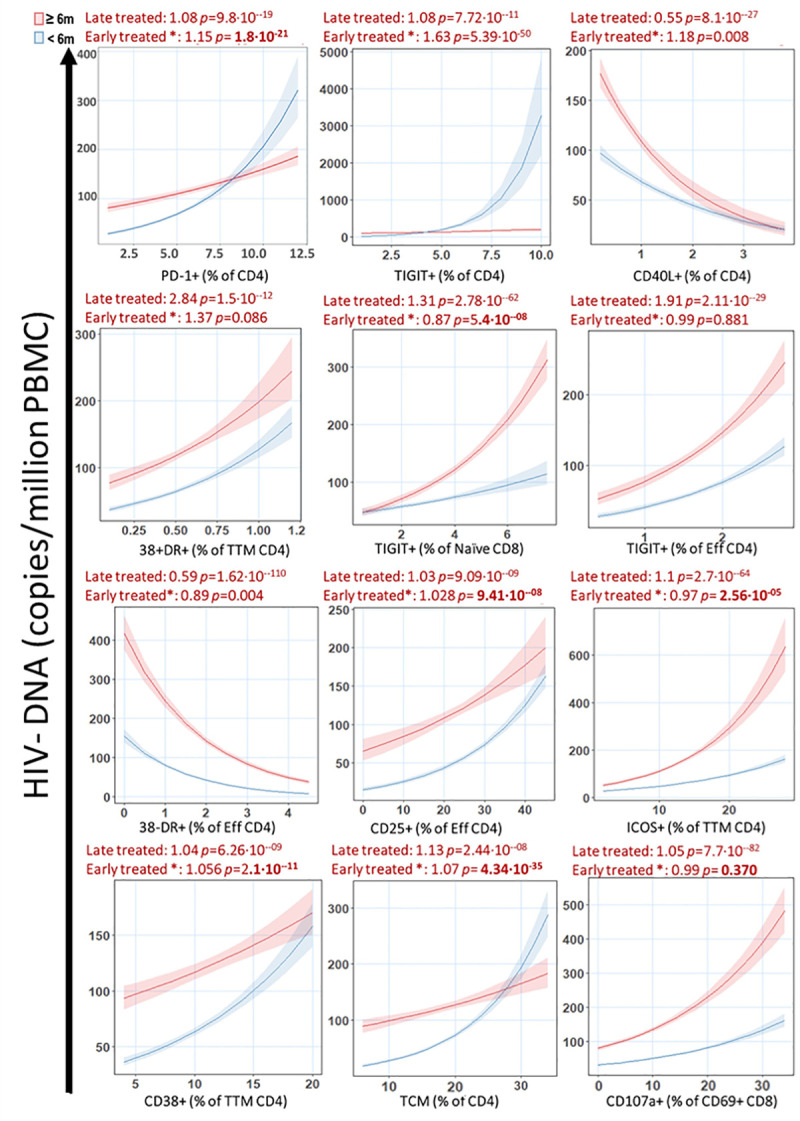
Predictors of HIV-1 DNA reservoir in participants treated before or after 6 months of age. After dividing the cohort in participants treated before 6 months of life (Blue) or after 6 months of life (Red), Predictors identified by penalized regression were plotted against predicted outcome (HIV-DNA) as in Fig 2. A value of effect above 1 indicates a positive contribution while a value between 0 and 1 indicates a negative contribution. *: effect of the association between the parameter and the reservoir when early treated compare to the late treated.

Due to the importance of the time of treatment initiation on preservation of immune functionality [35–36] and HIV viral reservoir size [31,37], we determined the statistical interaction of the variable TI on the calculated effect for all predictors. Among the predictors identified, all showed positive associations with HIV DNA, except for CD4/CD40L+ and CD4 Effector/CD38-HLA-DR+ which were negatively associated (**Fig 2**). Despite no significant univariate correlation between individual predictors and TI (**Fig 1**), the effect of the predictor on reservoir size was significantly impacted by TI in 8 out of 12 variables (**Fig 2**). To have a clearer understanding of the effect of TI on the effect of the predictors, we divided the cohort into individuals that initiated ART before 6 months of age (n = 23) or later than 6 months (n = 11). We observed that CD8 Naive/TIGIT+ and CD4 TTM/ICOS+ showed significantly stronger effect in individuals that initiated treatment later than 6 months of age, however CD4/TIGIT+, CD4/PD-1+, CD4 Effector/CD25+, CD4 TTM/CD38+ and TCM CD4 T cells showed a stronger association with HIV DNA in individuals that started treatment younger than 6 months of age (**Fig 3**). CD4/CD40L+ T cells retained the negative association with HIV DNA seen in the previous analysis in both groups, but it was stronger in individuals that started ART after 6 months of age. CD4 Effector/CD38-HLA-DR+ retained the negative association in both groups as well, but it was stronger for participants treated before 6 months of age.

### Immunologic variables are able to classify participants in 3 distinct clusters characterized by viral reservoir size

**Fig 4:**
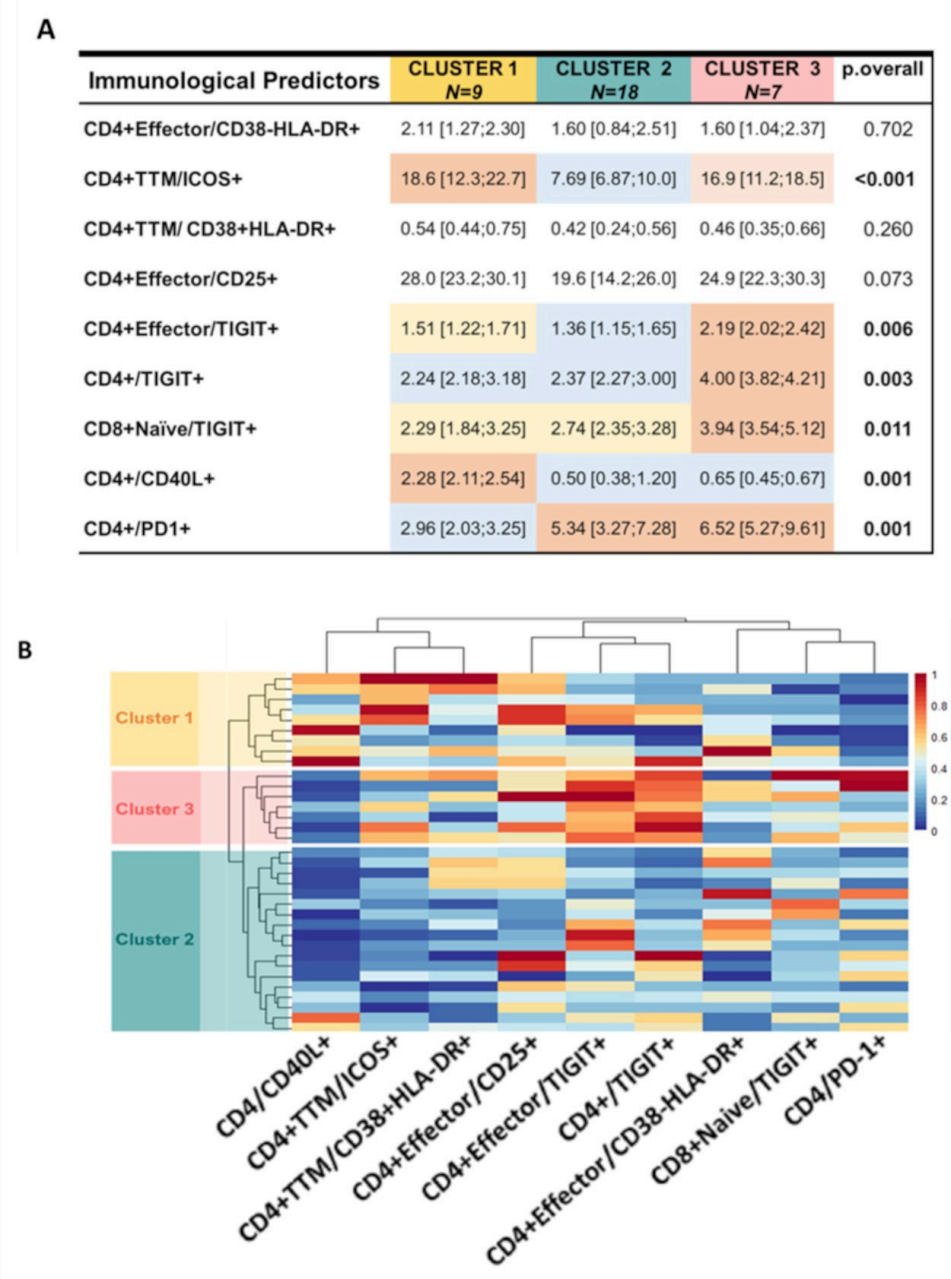
Immunological predictors identified by penalized regression could separate the cohort into 3 distinct clusters. **A) Table summarizing the 3 clusters identified by Ward’s Minimum Variance Clustering Method.** Number of participants assigned in each cluster is reported in the first row. Median and interquartile ranges of the frequencies of the immune markers are indicated within every cell and were compared among the 3 clusters (P value is reported in the far-right column). Frequencies are color coded from higher (**Red**) to lower (**Blue**). P value was calculated using Mann-Whitney U test. **B**) **Graphical representation of the frequencies of the predictors by heatmap graph.** Each column shows the frequency of one predictor identified by the label at the bottom while each row show the frequency of the predictors in one participant.

The next step of our approach was to validate that the predictors identified could discriminate between individuals with high and low reservoir size (as defined by the range observed in study participants shown in **Table 1**). Based on the frequencies of immune parameters identified by the Poisson regression, we used the Ward’s Minimum Variance Clustering Method to divide our cohort in clusters in an unsupervised fashion. We identified a 9-marker signature able to divide our cohort in 3 clusters (**Fig 4A and 4B**): Cluster 1 was characterized by the lowest level of CD4/PD-1+ and CD4/TIGIT+ T cells as well as the highest level of CD4/CD40L+ T cells. Cluster 2 showed frequencies of CD4/TIGIT+ T cells lower than cluster 3. It was also characterized by low CD4/CD40L+ and high CD4/PD-1+ T cells compared to cluster 1 but at the same level with cluster 3. Finally, cluster 3 had the highest expression of CD4/TIGIT+ T cells, highest level of CD4/PD-1+ T cells and a frequency of CD4/CD40L+ T cells lower than cluster 1 but at similar level of cluster 2.

**Fig 5:**
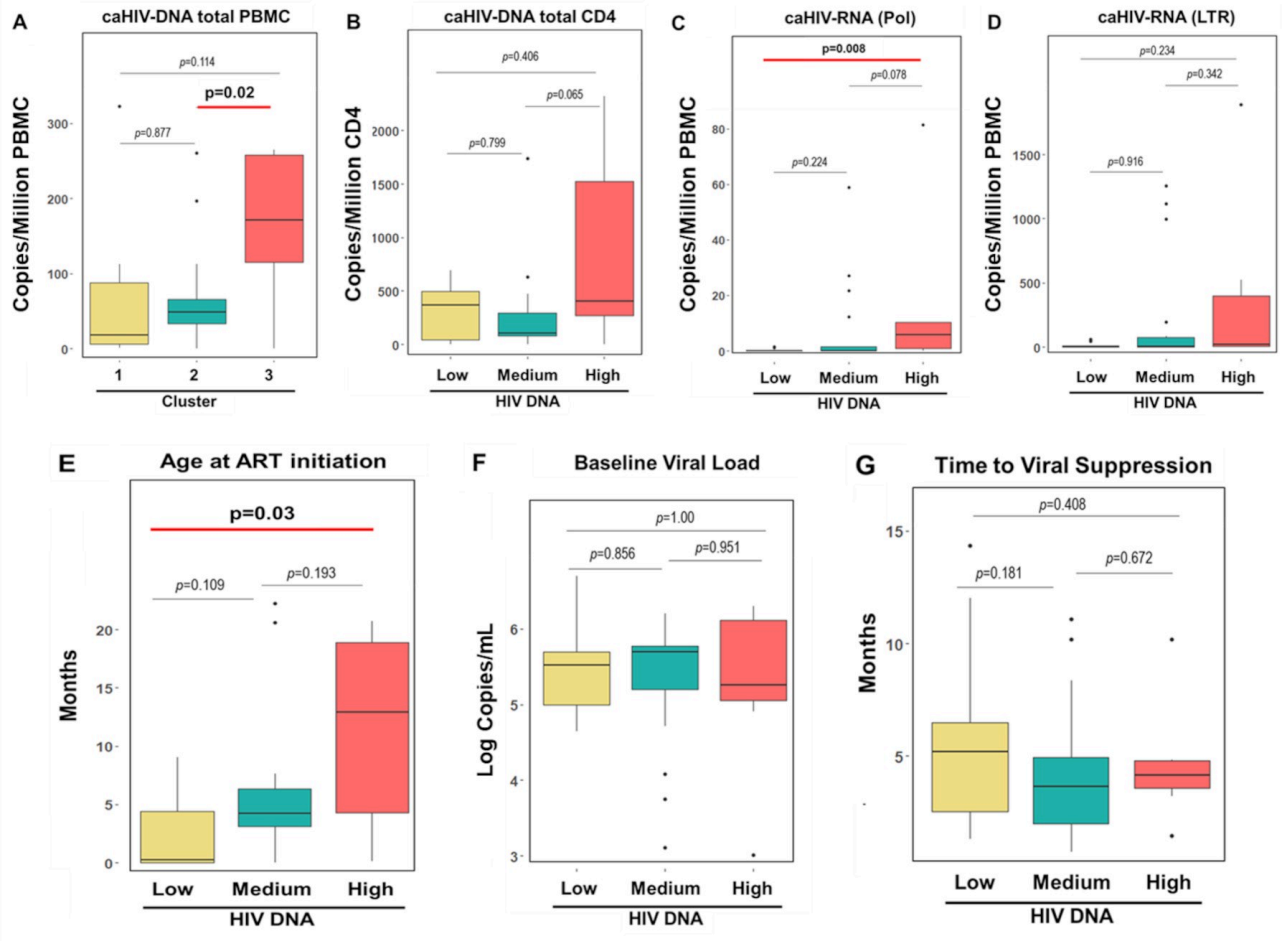
Clinical and virological characteristics of the 3 clusters based on the immunological predictors identified by the penalized regression. **Evaluation of: A) HIV DNA copies per million PBMC in the 3 clusters.** Clusters are depicted in yellow (cluster 1), green (cluster 2) and red (cluster 3). Based on the distribution of HIV DNA in the 3 clusters we renamed them in: Low (cluster 1), Medium (cluster 2) and High (cluster 3) HIV DNA, **B) HIV DNA copies per million CD4 T cells in the 3 clusters, C) unspliced cell associated HIV RNA (pol), D) total cell associated HIV RNA (LTR), E) Age at treatment initiation, F) Baseline viral load and G) Time to achieve viral suppression.** Box and whiskers graphs were chosen to show median and interquartile. Outliers are also shown using black dots. P values for the significant comparison are reported in bold.

We confirmed that the signature was associated with size of the reservoir measured by HIV DNA (**Fig 5A**) and we renamed the clusters based on the number of copies of HIV DNA/million PBMC as Low (Cluster 1), Medium (Cluster 2) and High (Cluster 3) HIV DNA. To further characterize the clinical and virological profile of these 3 clusters, we evaluated cell associated HIV RNA which was excluded from the original modelling since it was not performed in the total cohort. We found that high HIV DNA cluster also showed high cell associated HIV RNA, cell associated HIV DNA in CD4 T cells, and later ART initiation than the other clusters. Low and Medium HIV DNA clusters instead were similar in their profile but for a trend toward later ART initiation for Medium cluster. No differences in the time to reach viral suppression and viral load at ART initiation (**Fig 5B–5G**) or in viral dynamic (rebound, virological failure and spike) were observed in the 3 clusters (**S2 Table**).

In summary, higher proportions of PD-1+ CD4 T cells and TIGIT+ CD4 T cells along with low frequency of HIVsp (CD40L+) CD4 T cells were associated with delayed ART initiation and a larger viral reservoir (both DNA and cell associated RNA).

### CD4 and CD8 T cell functions negatively associate with frequency of PD-1+ CD4 T cells

**Fig 6:**
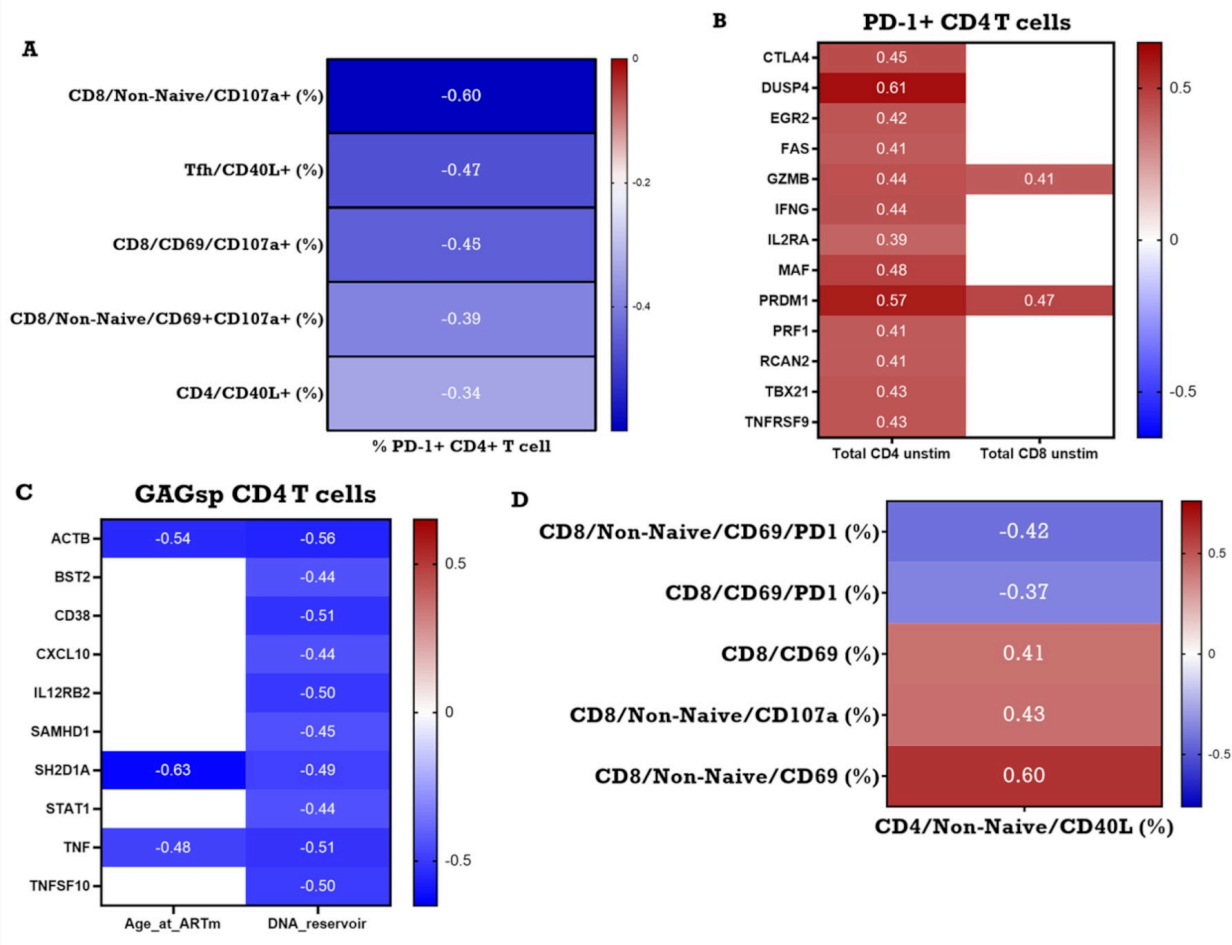
**A)** Association between CD4 and CD8 T cell functionality and baseline frequency of PD-1+ CD4 T cells. **B)** Association between frequency of PD-1 expressing CD4 T cells and gene expression in total CD4 and CD8 T cell. **C)** Association between Size of the reservoir and time of ART initiation with gene expression in HIVsp CD4 T cell. **D)** Association between frequency of HIVsp CD4 T cells and functionality of HIVsp CD8 T cell. Non-significant correlations are reported in white. R value for every significant correlation is color coded with negative r value reported in blue while positive are reported in red. The darker the color the stronger the correlation. The actual R value is also reported inside every square only for the significant correlations.

ICP molecules such as PD-1 and TIGIT are co-inhibitory receptors and they are known to be upregulated during T cell activation to prevent hyper-immune activation. Based on the observations that individuals with higher copies of cell associated HIV DNA also exhibited higher frequency of PD-1+ cells and lower frequency of HIVsp CD4 T cells and on the recent work from Macatangay and colleagues [38] that showed correlation between level of PD-1 expression on CD4 and CD8 T cells and GAG-specific IFNγ Elispot response, we hypothesized that PD-1 could have a direct association with HIVsp CD4 and HIVsp (CD69+) CD8 T cell frequencies and/or functions. To evaluate this hypothesis, we performed univariate correlation analysis between PD-1 expressing CD4 T cells and HIVsp T cell frequencies and functions in both CD4 and CD8 T cells. PD-1+ CD4 T cells were found to be negatively correlated to HIVsp total CD4 T cell frequencies. Moreover, these cells also negatively correlated with HIVsp CD8 T cells expressing CD107a, a marker of degranulation also considered a surrogate marker for cytotoxic CD8 T cells (**Figs 1 and 6A**).

Using multiplexed RT-PCR, we assessed the expression of 96 genes related to T cell immune activation, TCR signaling, and immune exhaustion in flow sorted HIVsp CD4 T cells but we found no correlation with frequency of PD-1+ CD4 T cells. Since in our model TIGIT expression was also a predictor for high HIV DNA, we included this marker as well in our analysis. We found that the frequency of TIGIT+ CD4 T cells was positively associated with the expression of genes related to senescence (DUSP4), HIV replication (EEF1A1), and impaired CD4 response (KLRG1) in HIVsp CD4 T cells (**S3 Fig**).

Since PD-1 expression on CD4 T cells in HIV treated individuals is also considered a hallmark of immune activation/exhaustion [39], we extended our investigation of the effect of high frequency of PD-1+ CD4 T cells on genes expressed in unstimulated flow sorted CD4 T cells and CD8 T cell (**Fig 6B**). We found PD-1 expressing CD4 T cells to associate with a greater number of genes in CD4 T cells than CD8 T cells (14 vs 2 genes, respectively). PD-1 expressing T cells were overall associated with a CD4 T cell molecular profile skewed toward Th1 type as demonstrated by the positive relation with TBX21 (T-BET), PRDM1 (BLIMP-1) and IFN-γ. Moreover, we also found that PD-1 expression on CD4 T cell associated with a more cytotoxic molecular profile with higher expression of GZMB (Granzyme B) and PRF1 (Perforin) genes. We also found a positive correlation with CTLA4, TNFRSF9 (4-1BB), and FAS gene expression indicating a more exhausted profile.

Overall, higher frequencies of PD-1 expressing CD4 T cells was associated with lower frequencies of HIVsp CD4 T cells and degranulating HIVsp CD8 T cells. Additionally, frequencies of PD-1 expressing CD4 T cells was associated with CD4 T cell transcriptional background skewed towards exhausted Th1 CD4 T cell profile at a resting state, while frequencies of TIGIT expressing CD4 T cells were associated with an impaired immune response in antigen-stimulated HIVsp CD4 T cells.

Since HIVsp CD4 T cells are important for direct antiviral activity and for supporting the HIVsp CD8 T cell response [40–41], we examined the relationship of HIVsp CD4 T cells with HIV reservoir. We found that higher expression of key genes of antiviral functionality (e.g. TNF, SAMHD1, STAT1 CXCL10) and immune activation (e.g. CD38, IL12RB2) in purified HIVsp CD4 T cell were associated with a lower size of HIV reservoir (**Fig 6C**). Results from univariate correlation analysis in our cohort showed that HIVsp CD4 T cells positively associate with the frequency of HIVsp CD8 T cells and degranulating CD8 T cells (**Fig 6D**). These findings support a relationship between frequency of HIVsp CD4 T cells and HIVsp CD8 T cells.

## Discussion

In this study we characterized the immune activation and immune checkpoint expression profiles of both CD4 and CD8 T cells along with frequency and function of HIV-specific CD4 and CD8 T cells in a cohort of children and adolescents vertically infected with HIV. For the first time, three different characteristics of T cell immunity known to be important for the persistence of the HIV viral reservoir [12–14] were evaluated together in a pediatric cohort. Out of the hundreds of markers studied, we identified a 9-marker immune signature predictive of HIV reservoir size. Within the signature variables, PD-1+ CD4 T cells, TIGIT+ CD4 T cells, and HIV-specific CD4 T cells were especially important in discriminating between individuals in our cohort that had a higher or lower number of cell associated HIV DNA copies.

To measure the size of the viral reservoir, we performed quantitative PCR of the total cell associated HIV DNA. The major drawback of this methodology is the inability of discern between replication competent and defective proviruses. Indeed, the field is now moving towards newer assays that were either not available at the time when the study was conducted (e.g. IPDA) [42] or were not feasible due to limited cell availability for the type of assay (e.g. QVOA) [43], both of which allow for assessment of intact vs defective provirus to some extent. Despite this drawback, the reliability of this simple but powerful assay to estimate the size of HIV reservoir is still well recognized in the field [33–34,44].

PD-1 expressing CD4 T cells and TIGIT expressing CD4 T cells are known to be associated with HIV viral reservoir in children [38] and adults especially when expressed simultaneously [13,23,28]. Despite sample availability limiting our ability to assess cells co-expressing these molecules, we found that these subsets retain their association and importance in pediatric populations as well. Additionally, we observed the frequency of HIV-specific CD4 T cells to be negatively associated with the number of copies of cell associated HIV DNA.

Due to the importance of early treatment initiation on size of viral reservoir [30,37], preservation of HIV-specific T cell immune response [5,16,35,45], and reduced immune activation, immune exhaustion and better immune reconstitution [44,46–47], we evaluated the effect of time of ART initiation, in this case age of the child in months, on the immune predictors for HIV DNA copies identified in our cohort. We observed that time of treatment initiation has a strong effect on many of these immune variables. Interestingly, when we divided our cohort based on time of ART initiation, we found that a higher frequency of PD-1+ or TIGIT+ CD4 T cell had a greater effect on the reservoir size in participants who had TI within 6 months of age, while the importance of HIV-specific CD4 T cells as predictors of lower HIV reservoir was greater in participants with delayed TI, after age 6 months. Notably, high frequency of PD-1 or TIGIT expressing CD4 T cell were predictors of high HIV reservoir especially in participants treated within 6 months. In this group in fact, their effect on size of HIV reservoir was increased by 15% and 63% for PD-1 and TIGIT, respectively. This result suggests that in HIV infected infants treated earlier than 6 months of age, CD4 T cells expressing these 2 markers could have a more important role on HIV persistence compared to individuals treated later [13,23,28].

Confirming the importance of these 3 predictors (PD-1+ CD4 T cell, TIGIT+ CD4 T cell and HIV-specific CD4 T cells), the unsupervised clustering method also showed they were related with cluster 3 that had the highest proportion of PD-1+ CD4 T cells and TIGIT+ CD4 T cells, the lowest frequency of HIV-specific CD4 T cells, the largest viral reservoir (both DNA and cell associated RNA), and delayed ART initiation. We also evaluated whether the 3 clusters exhibited different viral dynamics (e.g. time to viral suppression, virological failure) but we found no significant differences. The only other subsets differentiating the 3 clusters significantly were Effector CD4 T cells expressing TIGIT, Naïve CD8 T cells expressing TIGIT and transitional memory CD4 T cells expressing the activation marker ICOS. TIGIT expressing subsets were always higher in cluster 3 compared to cluster 1 and 2, following the same pattern already observed for total CD4 expressing TIGIT or PD-1, reinforcing the idea that a higher reservoir size is accompanied by higher T cell exhaustion. On the other hand, transitional memory CD4 T cells expressing the activation marker ICOS showed a different pattern; in fact, it was high in both, cluster 1 and cluster 3, compared to cluster 2.

Taking advantage of the extensive phenotypic analysis and multiplexed real time PCR performed on flow sorted HIV-specific and total CD4 T cells in this cohort, we also investigated how these subsets interact with each other. We found evidence that these subsets are related to each other and to the viral reservoir size, however we were unable to fully unravel the mechanism of this interaction. In fact, our data suggest a link between the lower frequency of HIV-specific CD4 T cells and a molecular profile of exhaustion that could prevent the activation (and thereby detection in our system) of these cells. Additionally, based on our results we cannot discriminate whether TIGIT expression on CD4 T cells has a direct effect on molecular profiles of HIV-specific CD4 T cells or indirect due to higher overall exhaustion as determined by PD-1+ CD4 T cell frequencies associated with exhausted Th1 gene expression in total CD4 T cells. There is also the possibility that both these scenarios are occurring simultaneously. These results are even more intriguing in the light of the observation that the relationship between the copies of HIV DNA and the expression of PD-1 or TIGIT in CD4 T cells and the frequency of HIV-specific CD4 T cells are differently affected by time of treatment initiation, suggesting that an early intervention could have a more extensive role than previously appreciated, not only limiting seeding of the reservoir but potentially affecting the mechanisms behind persistence.

An additional point of discussion is whether HIV-specific CD4 T cells affect the size of the viral reservoir directly or indirectly. We found that size of the viral reservoir is negatively associated with antiviral molecular profile in HIV-specific CD4 T cells suggesting a possible direct mechanism but we also observed that HIV-specific CD4 T cells correlate with both frequency and function of HIV-specific CD8 T cells. Multiple lines of evidence in the literature support the important role of CD4 T cells in promoting CD8 T cell function during viral infection [48–51] and in rescuing CD8 T cells from exhaustion in the context of chronic infection [52]. Exploring the direct/indirect effect of HIV-specific CD4 T cells on the infected cells was beyond the scope of this work and we could not perform the functional experiments needed to validate this hypothesis.

Our study was limited by sample availability such that we could only perform a cross-sectional evaluation in children and adolescents, at timepoints which were often several years after treatment initiation. Nevertheless, the findings expand the role of time of treatment initiation on the size of HIV reservoir showing that time of ART initiation may have long lasting effects not only on functionality of HIV-specific CD4 and CD8 T cells [35] or on the size of HIV viral reservoir [31] but also on the immune predictors of the size of HIV reservoir. Additionally, despite having performed our analysis on 34 individuals, validation of our immunological signature will require larger cohorts.

In conclusion, we identified a T cell immune signature associated with size of viral reservoir in a pediatric population, that is currently lacking in the field. Frequencies of PD-1+ and TIGIT+ CD4 T cells along with the frequency of HIV-specific CD4 T cells were at the core of this immune signature and able to discriminate between individuals with low or high viral reservoir. This signature could be used to focus interventions toward relevant targets or as an endpoint for testing the efficacy of therapies aimed to achieve HIV remission in pediatric populations.

## Methods

### Ethics statement

This was a multicenter, cross-sectional study enrolling subjects from 7 European pediatric HIV clinical research centers contributing to EPIICAL, in England (3), Spain (2), and Italy (2). The CARMA study was approved by: bambino Gesù Children Hospital ethical committee Prot. n. 1387; UK National Research Ethics Board REC Reference: 17/LO/1182, IRAS Project ID: 227799; CEIm Hospital Universitario 12 de Octubre and CEIm Hospital Universitario Gregorio Marañon Code 17/028. Written informed assent/consent obtained from adolescents and/or parent(s)/legal guardian in accordance with country-specific law. For Padua, leftover blood samples from routine clinical analysis were used. No IRB was needed but a specific informed consent was collected. Each participant received a unique study number, under which data were pseudo-anonymized.

### Human subjects

PBMC were received from 34 perinatally HIV infected children and adolescents (age range 4.4–19.1 years) from the CARMA cohort [31]. All study participants had durable viral control (HIV RNA load <50copies/mL) for a median of 5 years. Characteristics of the cohort are reported in **Table 1**.

### Flow cytometry

Cryopreserved PBMC were thawed and rested overnight at 37°C 5% CO2. Cells were stained directly for ex vivo evaluation of immune activation or immune checkpoint markers or stimulated for the evaluation of CD4 and CD8 T cell functionality. Data were collected for total CD4 and CD8 T cell as well as within maturational subsets: Naïve (CD27+CD45RO-), Central Memory (CD27+CD45RO+CCR7+), Transitional Memory (CD27+CD45RO+CCR7-), Effector Memory (CD27-CD45RO+) and Effector (CD27-CD45RO-) for CD8 T cell. Naïve (CD27+CD45RO-), Central Memory (CD27+CD45RO+CCR7+), Transitional Memory (CD27+CD45RO+CCR7-), Effector Memory (CD27-CD45RO+), Effector (CD27-CD45RO-) and peripheral T follicular helper cells (Tfh, CD27+CD45RO+CXCR5+) for CD4 T cell. (**S1 Fig**). Despite some studies include PD-1 as a marker for the gating of Tfh, we excluded it based on multiple studies which have shown that circulating Tfh exhibit much lower PD-1 expression compared to lymph node resident CXCR5+ CD4 T cells [53–55] and that PD-1 expression is altered in HIV infected individuals [56].

#### mAb flow cytometry

The following fluorochrome-conjugated anti-human mAb were used for flow cytometry studies: LAG3 BV650, TIGIT PE-Cy7, CTLA4 PE, CD25 PE-Dazzle 594, TIM3 PerCP-Cy5.5, CD19 Alexa Fluor 700, CCR4 BV421, ICOS PE-Cy7, CCR5 PE-Dazzle 594, HLA-DR PE, CCR7 FITC, CD38 BV711, PD-L1 BV711, CCR6 Alexa Fluor 700, PD-1 BV421, CD40L BV605, perforin PE-Dazzle 594, and CD8 PerCP from BioLegend (San Diego, CA); CD3 BUV496, CD4 APC-Cy7, CD4 APC-H7, CD127 BV605, Ki-67 PerCP-Cy5.5, PD-1 BV650, CXCR3 BV605, CD69 BV650, IL-2 BV711, CXCR5 Alexa Fluor 647, IFN-γ PE-Cy7, TNF-α FITC, granzyme B Alexa Fluor 700 and CD27 BV480 from BD Biosciences (San Jose, CA); IL-21 PE from eBioscience, (San Diego, CA); and CD45RO PE-Cy5.5 from Beckman Coulter (Fullerton, CA). LIVE/DEAD Fixable Blue Dead Cell Stain Kit from Thermo Fisher Scientific (Boston, MA) was used to detect and exclude dead cells. All the reagents were tested and titrated for optimum concentration before usage.

#### PBMC stimulation and flow cytometry staining

Cells were stimulated at the concentration of 5 million per milliliter for 12 h in presence of 2 μg/ml GAG PTE peptides (AIDS Reagent Program, Division of AIDS, National Institute of Allergy and Infectious Diseases [NIAID], National Institutes of Health [NIH]: HIV-1 PTE Gag Peptide Pool from NIAID, Division of AIDS), 1 μg/ml of staphylococcal enterotoxins B (SEB; List Biological Laboratories) or medium (negative control). At the beginning of culture, the following reagents were added: 1 μg/ml costimulatory molecule anti-CD28 mAb (BD Biosciences), 1 ug/mL of anti-CD49d (BD Biosciences), 0.65 μl/ml protein transport inhibitor monensin (BD GolgiStop), and the degranulation marker (CD107a). After 6 h of stimulation, brefeldin A (10 μg/ml) was added to the stimulation to permit the accumulation of cytokines within the PBMC [57].

Following the culture period, cells were treated with Human TruStain FcX (Biolegend, San Diego CA) for blocking the FcR-mediated Ig Fc binding. Cells were stained for flow cytometry using previously titrated mAb. Live cells were stained with Fixable Blue LIVE/DEAD and then incubated with appropriate surface Ab mixture (CD3, CD4, CD8, CD27, CD45RO, and CXCR5). Cells were then fixed and permeabilized with Cytofix/Cytoperm Buffer (BD Biosciences) and stained for intracellular molecules CD69, CD40L, IL-2, IFN-γ, TNF-α, IL-21, granzyme B, and perforin.

Total CD4 and CD8 T cells and subsets were analyzed for the expression of activation induced molecule CD40L or CD69 to identify the Ag-specific T cells. Ag-specific cells were further evaluated for the stimulus-induced expression of intracellular cytokines IFN-γ, IL-21, IL-2, and TNF-α for CD4 T cells and IFN-γ, IL-2, TNF-α, perforin, and granzyme B for CD8 T cells. Stained cells were acquired on a BD LSRFortessa (BD Biosciences) and analysis performed using FlowJo v10.0.8 (Tree Star) software. In order to identify expression of individual markers, we used a gating control containing only lineage markers to identify the positivity of the markers (e.g. CD40L, CD69, etc) and an unstimulated control with no antigen (medium only) to gate for cells responding to the stimulation (**S4 Fig**).

### RT-PCR

#### AIM Assay and sorting for transcriptomic analysis

Cryopreserved PBMC were thawed and allowed to rest for 2–3 hours prior to stimulation with HIV gag PTE peptides for a modified AIM (Activation Induced Molecule) assay [58]. Cells were cultured at 5 million per milliliter for 18–20 h in presence of 2 μg/ml GAG PTE peptides (AIDS Reagent Program, Division of AIDS, National Institute of Allergy and Infectious Diseases [NIAID], National Institutes of Health [NIH]: HIV-1 PTE Gag Peptide Pool from NIAID, Division of AIDS), 1 μg/ml of staphylococcal enterotoxin B (SEB; List Biological Laboratories) or medium (negative control). At the beginning of culture, the following reagents were added: 1 μg/ml costimulatory molecule anti-CD28 mAb (BD Biosciences), 1ug/mL of anti-CD49d (BD Biosciences) and 0.5ug/mL the degranulation marker (CD107a PerCP-Cy5.5).

Following the culture period, cells were stained for flow sorting using previously titrated mAb (CD3, CD4, CD8, OX40, and CD25). Sorting was performed using the gating strategy shown (**S5 Fig**) and 500 cells were collected per sorting population for downstream gene expression analysis.

#### Fluidigm

Sorted cells were subject to specific target preamplification (STA) using CellsDirect One-Step qRT-PCR Kit, followed by Exonuclease I treatment.

High-throughput real-time PCR was run on Fluidigm Biomark HD System (Fluidigm, South San Francisco, CA, USA) using 96.96 Dynamic Array Integrated Fluidic Circuit (IFC). This approach was used to evaluate the expression of 96 genes (**S3 Table**) in our samples.

### Viral reservoir measurements

#### HIV cell–associated DNA in total PBMC

Total HIV DNA was quantified from PBMC at the enrollment time point for all patients using an in-house, real-time quantitative PCR assay as previously described [31]. Briefly, total nucleic acid was extracted from PBMCs on the Qiasymphony platform using the DSP virus/pathogen mini kit (Qiagen) according to the manufacturer’s protocol. An ABI PRISM 7500 real-time PCR instrument with Invitrogen RT-PCR reagents was used for amplification and detection of HIV-1 DNA. The HIV DNA copies were calculated as HIV-1 copies per 10^6 haploid pyruvate dehydrogenase (PDH) copies. Results are reported as copies of HIV per million cells [59].

#### HIV cell–associated DNA in CD4 T cells

CD4 cell subset were isolated from PBMC with CD4+ T Cell Isolation Kit (Miltenyi Biotec, Auburn, California, USA) following manufacturer’s instructions. The purity of the enriched fraction (>90%) was checked for a selection of samples with flow cytometry by staining with fluorescent-conjugated antibodies CD3-FITC and CD4-PerCP. HIV-1 DNA levels in separated CD4 T cells were measured by the QX200 Droplet Digital PCR (ddPCR) system (Bio-Rad, Pleasanton, CA), using the LTRfw and LTRrv primers [60]; the HIV-1 copy number was normalized against the TERT copy number, and the final results were expressed as HIV-1 DNA copies/10^6 CD4 cells.

#### HIV cell–associated RNA in total PBMC

Briefly, total nucleic acids were extracted from PBMCs on the Qiasymphony platform using the DSP virus/pathogen mini kit (Qiagen) according to the manufacturer’s protocol. For qRT-PCR of total (LTR) and unspliced (pol) ca-RNA primers of previously reported assays were used [59,61]. The HIV caRNA was normalized against TBP1 and IPO8 expression in an in-house assay.

### Statistical analysis

For the flow cytometry and RT-PCR data, all correlations were evaluated using the Spearman’s rank correlation coefficient test. Comparison between two groups was made using the Mann–Whitney U test. Statistical tests were two sided, and the tests were considered statistically significant with a p value ≤0.05 and performed using GraphPad Prism version 8.0.0 for Windows (GraphPad Software, La Jolla, CA, http://www.graphpad.com).

To describe the association between the cell subsets and HIV reservoir, we performed a penalized elastic net multivariable Poisson regression. The model included age at ART (also called time of treatment initiation), baseline viral load, baseline CD4 percentage, and the age at HIV reservoir measurement as confounders. The age at ART groups (early treated, late treated) or age at ART (months) was included as interaction factor for additional analysis (**Fig 3**). Overdispersion was checked using the dispersion test implemented in AER R Package (https://CRAN.R-project.org/package=AER) and zero-inflation using a zero score test [62]. A hierarchical clustering analysis was performed to identify subphenotypes. Pearson correlation was performed as distance matrix and Ward’s D2 as clustering method. To determine the optimal number of clusters we run 30 indices implemented in NbClust R package (http://www.jstatsoft.org/v61/i06) and select the majority vote for best partition. All these analyses were performed using R (R Core Team (2014). R: A language and environment for statistical computing. R Foundation for Statistical Computing, Vienna, Austria. URL http://www.R-project.org/).

## Supporting information

S1 Table Phenotypic markers included in the model. (DOCX)

S2 Table Virological characteristics of the clusters. (DOCX)

S3 Table List of the genes studied. (DOCX)

S1 Fig Gating strategy and distribution of the markers analysed by flow cytometry. **A)** Example of gating strategy used for identification of the different CD4 and CD8 Maturational subsets.
(TIF)

S2 Fig List of the markers/cytokines evaluated in the 3 different flow panels: Immune Activation, Immune Checkpoint and T cell functions (evaluated after peptides stimulation). **A-B)** Distribution of the maturational subsets in CD4 **(A)** and CD8 **(B)** T cells. **C-D)** Distribution of the immune activation and immune checkpoint markers expression in total CD4 **(C)** and CD8 **(D)** T cells. **E-F)** Distribution of cytokines’ expression in HIV specific (CD40L+) CD4 **(E)** and HIV specific (CD69+) CD8 **(F)** T cells after stimulation with HIV GAG PTE peptides.
(TIF)

S3 Fig Association between frequency of PD-1 or TIGIT expressing CD4 T cells with gene expression in GAGsp CD4 T cell. Non-significant correlations are reported in white. R value for every significant correlation is color coded with negative r value reported in blue while positive are reported in red. The darker the color the stronger the correlation. The actual R value is also reported inside every squares only for the significant correlations.
(TIF)

S4 Fig Gating Strategy for AIM Assay for flow cytometry. Gating control (**FMO**) was set to define the negative population. An unstimulated condition (**Neg**) and a stimulation using polyclonal stimulus SEB as positive control (**Pos**) were used to identify the cells responding to the stimulation.
(TIF)

S5 Fig Gating Strategy for AIM Assay and Cell Sorting for Fluidigm BioMark Gene expression Analysis. Numbered populations were sorted: 1 and 2 were from the medium (unstimulated) condition, 3 and 4 from the Gag-stimulated condition, and 5 and 6 from the SEB stimulated condition.
(TIF)
