# Supplementary material for: T cell immune discriminants of HIV reservoir size in a pediatric cohort of perinatally infected individuals
Source: PLoS Pathog. 2021 Apr 26;17(4):e1009533. doi: 10.1371/journal.ppat.1009533 (PMC8112655; doi:10.1371/journal.ppat.1009533)
Supplement: S1 Table — (DOCX) [file ppat.1009533.s001.docx]

**Supplementary Table 1:**

| **Immune activation Panel** | **ICP Panel** | **Stimulation Panel** |
| --- | --- | --- |
| T cell/CD4 (%) | CD4 (%) | CD4 (%) |
| CD4/Effector (%) | CD4/CD25 (%) | CD4/CD40L (%) |
| CD4/Effector/CCR5+ (%) | CD4/CXCR5 subset (%) | CD4/CD40L/CD69 (%) |
| CD4/Effector/CD38+ (%) | CD4/LAG3 (%) | CD4/CD40L/IFNg (%) |
| CD4/Effector/CXCR5+ (%) | CD4/PD1 (%) | CD4/CD40L/IL2 (%) |
| CD4/Effector/HLA-DR+ (%) | CD4/PDL1 (%) | CD4/CD40L/IL21 (%) |
| CD4/Effector/ICOS+ (%) | CD4/Naive/CD25 (%) | CD4/CD40L/PD1 (%) |
| CD4/Effector/Ki67+ (%) | CD4/Naive/CXCR5 subset (%) | CD4/CD40L/TNF (%) |
| CD4/Effector/Q9: CD38- , HLA-DR+ (%) | CD4/Naive/LAG3 (%) | CD4/CD69 (%) |
| CD4/Effector/Q10: CD38+ , HLA-DR+ (%) | CD4/Naive/PD1 (%) | CD4/IFNg (%) |
| CD4/Effector/Q11: CD38+ , HLA-DR- (%) | CD4/Naive/PDL1 (%) | CD4/IL2 (%) |
| CD4/Effector/Q12: CD38- , HLA-DR- (%) | CD4/Naive/TIGIT (%) | CD4/IL21 (%) |
| CD4/CCR5+ (%) | CD4/Naive/Tim3 (%) | CD4/Mature (%) |
| CD4/CD38+ (%) | CD4/Q2: CD45RO+ , CD27+/TCM/CD25 (%) | CD4/Mature/CD40L (%) |
| CD4/total Memory (%) | CD4/Q2: CD45RO+ , CD27+/TCM/CXCR5 subset (%) | CD4/Mature/CD40L/CD69 (%) |
| CD4/CXCR5+ (%) | CD4/Q2: CD45RO+ , CD27+/TCM/LAG3 (%) | CD4/Mature/CD40L/IFNg (%) |
| CD4/HLA-DR+ (%) | CD4/Q2: CD45RO+ , CD27+/TCM/PD1 (%) | CD4/Mature/CD40L/IL2 (%) |
| CD4/ICOS+ (%) | CD4/Q2: CD45RO+ , CD27+/TCM/PDL1 (%) | CD4/Mature/CD40L/IL21 (%) |
| CD4/Ki67+ (%) | CD4/Q2: CD45RO+ , CD27+/TCM/TIGIT (%) | CD4/Mature/CD40L/PD1 (%) |
| CD4/Naive (%) | CD4/Q2: CD45RO+ , CD27+/TCM/Tim3 (%) | CD4/Mature/CD40L/TNF (%) |
| CD4/Naive/CCR5+ (%) | CD4/Q2: CD45RO+ , CD27+/Tfh/CD25 (%) | CD4/Mature/CD69 (%) |
| CD4/Naive/CD38+ (%) | CD4/Q2: CD45RO+ , CD27+/Tfh/LAG3 (%) | CD4/Mature/IFNg (%) |
| CD4/Naive/CXCR5+ (%) | CD4/Q2: CD45RO+ , CD27+/Tfh/PD1 (%) | CD4/Mature/IL2 (%) |
| CD4/Naive/HLA-DR+ (%) | CD4/Q2: CD45RO+ , CD27+/Tfh/PDL1 (%) | CD4/Mature/IL21 (%) |
| CD4/Naive/ICOS+ (%) | CD4/Q2: CD45RO+ , CD27+/Tfh/TIGIT (%) | CD4/Mature/PD1 (%) |
| CD4/Naive/Ki67+ (%) | CD4/Q2: CD45RO+ , CD27+/Tfh/Tim3 (%) | CD4/Mature/TNF (%) |
| CD4/Naive/Q9: CD38- , HLA-DR+ (%) | CD4/Q2: CD45RO+ , CD27+/TTM/CD25 (%) | CD4/PD1 (%) |
| CD4/Naive/Q10: CD38+ , HLA-DR+ (%) | CD4/Q2: CD45RO+ , CD27+/TTM/CXCR5 subset (%) | CD4/Q1: CD45RO- , CD27+ (%) |
| CD4/Naive/Q11: CD38+ , HLA-DR- (%) | CD4/Q2: CD45RO+ , CD27+/TTM/LAG3 (%) | CD4/Q2: CD45RO+ , CD27+ (%) |
| CD4/Naive/Q12: CD38- , HLA-DR- (%) | CD4/Q2: CD45RO+ , CD27+/TTM/PD1 (%) | Tfh (%) |
| CD4/Q2: CD45RO+ , CD27+ (%) | CD4/Q2: CD45RO+ , CD27+/TTM/PDL1 (%) | Tfh/CD40L (%) |
| CD4/Q2: CD45RO+ , CD27+/TCM \| Freq. of CD4 (%) | CD4/Q2: CD45RO+ , CD27+/TTM/TIGIT (%) | Tfh/CD40L/CD69 (%) |
| CD4/Q2: CD45RO+ , CD27+/TCM/CCR5+ (%) | CD4/Q2: CD45RO+ , CD27+/TTM/Tim3 (%) | Tfh/CD40L/IFNg (%) |
| CD4/Q2: CD45RO+ , CD27+/TCM/CD38+ (%) | CD4/TEM/CD25 (%) | Tfh/CD40L/IL2 (%) |
| CD4/Q2: CD45RO+ , CD27+/TCM/CXCR5+ (%) | CD4/TEM/CXCR5 subset (%) | Tfh/CD40L/IL21 (%) |
| CD4/Q2: CD45RO+ , CD27+/TCM/HLA-DR+ (%) | CD4/TEM/LAG3 (%) | Tfh/CD40L/PD1 (%) |
| CD4/Q2: CD45RO+ , CD27+/TCM/ICOS+ (%) | CD4/TEM/PD1 (%) | Tfh/CD40L/TNF (%) |
| CD4/Q2: CD45RO+ , CD27+/TCM/Ki67+ (%) | CD4/TEM/PDL1 (%) | Tfh/CD69 (%) |
| CD4/Q2: CD45RO+ , CD27+/TCM/Q9: CD38- , HLA-DR+ (%) | CD4/TEM/TIGIT (%) | Tfh/IFNg (%) |
| CD4/Q2: CD45RO+ , CD27+/TCM/Q10: CD38+ , HLA-DR+ (%) | CD4/TEM/Tim3 (%) | Tfh/IL2 (%) |
| CD4/Q2: CD45RO+ , CD27+/TCM/Q11: CD38+ , HLA-DR- (%) | CD4/Effector/CD25 (%) | Tfh/IL21 (%) |
| CD4/Q2: CD45RO+ , CD27+/TCM/Q12: CD38- , HLA-DR- (%) | CD4/Effector/CXCR5 subset (%) | Tfh/PD1 (%) |
| CD4/Q2: CD45RO+ , CD27+/Tfh (%) | CD4/Effector/LAG3 (%) | Tfh/TNF (%) |
| CD4/Q2: CD45RO+ , CD27+/Tfh \| Freq. of CD4 (%) | CD4/Effector/PD1 (%) | CD4/Q3: CD45RO+ , CD27- (%) |
| CD4/Q2: CD45RO+ , CD27+/Tfh/CCR5+ (%) | CD4/Effector/PDL1 (%) | CD4/Q4: CD45RO- , CD27- (%) |
| CD4/Q2: CD45RO+ , CD27+/Tfh/CD38+ (%) | CD4/Effector/TIGIT (%) | CD4/TNF (%) |
| CD4/Q2: CD45RO+ , CD27+/Tfh/HLA-DR+ (%) | CD4/Effector/Tim3 (%) | CD8 (%) |
| CD4/Q2: CD45RO+ , CD27+/Tfh/ICOS+ (%) | CD4/TIGIT (%) | CD8/CD69 (%) |
| CD4/Q2: CD45RO+ , CD27+/Tfh/Ki67+ (%) | CD4/Tim3 (%) | CD8/CD69/CD107a (%) |
| CD4/Q2: CD45RO+ , CD27+/Tfh/Q9: CD38- , HLA-DR+ (%) | CD8/CD25 (%) | CD8/CD69/GrzB (%) |
| CD4/Q2: CD45RO+ , CD27+/Tfh/Q10: CD38+ , HLA-DR+ (%) | CD8/CXCR5 subset (%) | CD8/CD69/IFNg (%) |
| CD4/Q2: CD45RO+ , CD27+/Tfh/Q11: CD38+ , HLA-DR- (%) | CD8/LAG3 (%) | CD8/CD69/IL2 (%) |
| CD4/Q2: CD45RO+ , CD27+/Tfh/Q12: CD38- , HLA-DR- (%) | CD8/PD1 (%) | CD8/CD69/PD1 (%) |
| CD4/Q2: CD45RO+ , CD27+/Tfh/Q17: CD38- , ICOS+ (%) | CD8/PDL1 (%) | CD8/CD69/Perforin (%) |
| CD4/Q2: CD45RO+ , CD27+/Tfh/Q18: CD38+ , ICOS+ (%) | CD8/Naive/CD25 (%) | CD8/CD69/TNF (%) |
| CD4/Q2: CD45RO+ , CD27+/Tfh/Q19: CD38+ , ICOS- (%) | CD8/Naive/LAG3 (%) | CD8/CD107a (%) |
| CD4/Q2: CD45RO+ , CD27+/Tfh/Q20: CD38- , ICOS- (%) | CD8/Naive/PD1 (%) | CD8/GrzB (%) |
| CD4/Q2: CD45RO+ , CD27+/TTM \| Freq. of CD4 (%) | CD8/Naive/PDL1 (%) | CD8/IFNg (%) |
| CD4/Q2: CD45RO+ , CD27+/TTM/CCR5+ (%) | CD8/Naive/TIGIT (%) | CD8/IL2 (%) |
| CD4/Q2: CD45RO+ , CD27+/TTM/CD38+ (%) | CD8/Naive/Tim3 (%) | CD8/Mature (%) |
| CD4/Q2: CD45RO+ , CD27+/TTM/CXCR5+ (%) | CD8/Q2: CD45RO+ , CD27+/TCM/CD25 (%) | CD8/Mature/CD69 (%) |
| CD4/Q2: CD45RO+ , CD27+/TTM/HLA-DR+ (%) | CD8/Q2: CD45RO+ , CD27+/TCM/LAG3 (%) | CD8/Mature/CD69/CD107a (%) |
| CD4/Q2: CD45RO+ , CD27+/TTM/ICOS+ (%) | CD8/Q2: CD45RO+ , CD27+/TCM/PD1 (%) | CD8/Mature/CD69/GrzB (%) |
| CD4/Q2: CD45RO+ , CD27+/TTM/Ki67+ (%) | CD8/Q2: CD45RO+ , CD27+/TCM/PDL1 (%) | CD8/Mature/CD69/IFNg (%) |
| CD4/Q2: CD45RO+ , CD27+/TTM/Q9: CD38- , HLA-DR+ (%) | CD8/Q2: CD45RO+ , CD27+/TCM/TIGIT (%) | CD8/Mature/CD69/IL2 (%) |
| CD4/Q2: CD45RO+ , CD27+/TTM/Q10: CD38+ , HLA-DR+ (%) | CD8/Q2: CD45RO+ , CD27+/TCM/Tim3 (%) | CD8/Mature/CD69/PD1 (%) |
| CD4/Q2: CD45RO+ , CD27+/TTM/Q11: CD38+ , HLA-DR- (%) | CD8/Q2: CD45RO+ , CD27+/TTM/CD25 (%) | CD8/Mature/CD69/Perforin (%) |
| CD4/Q2: CD45RO+ , CD27+/TTM/Q12: CD38- , HLA-DR- (%) | CD8/Q2: CD45RO+ , CD27+/TTM/LAG3 (%) | CD8/Mature/CD69/TNF (%) |
| CD4/Q9: CD38- , HLA-DR+ (%) | CD8/Q2: CD45RO+ , CD27+/TTM/PD1 (%) | CD8/Mature/CD107a (%) |
| CD4/Q10: CD38+ , HLA-DR+ (%) | CD8/Q2: CD45RO+ , CD27+/TTM/PDL1 (%) | CD8/Mature/GrzB (%) |
| CD4/Q11: CD38+ , HLA-DR- (%) | CD8/Q2: CD45RO+ , CD27+/TTM/TIGIT (%) | CD8/Mature/IFNg (%) |
| CD4/Q12: CD38- , HLA-DR- (%) | CD8/Q2: CD45RO+ , CD27+/TTM/Tim3 (%) | CD8/Mature/IL2 (%) |
| CD4/TEM (%) | CD8/TEM/CD25 (%) | CD8/Mature/PD1 (%) |
| CD4/TEM/CCR5+ (%) | CD8/TEM/LAG3 (%) | CD8/Mature/Perforin (%) |
| CD4/TEM/CD38+ (%) | CD8/TEM/PD1 (%) | CD8/Mature/TNF (%) |
| CD4/TEM/CXCR5+ (%) | CD8/TEM/PDL1 (%) | CD8/PD1 (%) |
| CD4/TEM/HLA-DR+ (%) | CD8/TEM/TIGIT (%) | CD8/Perforin (%) |
| CD4/TEM/ICOS+ (%) | CD8/TEM/Tim3 (%) | CD8/Q1: CD45RO- , CD27+ (%) |
| CD4/TEM/Ki67+ (%) | CD8/Effector/CD25 (%) | CD8/Q2: CD45RO+ , CD27+ (%) |
| CD4/TEM/Q9: CD38- , HLA-DR+ (%) | CD8/Effector/LAG3 (%) | CD8/Q3: CD45RO+ , CD27- (%) |
| CD4/TEM/Q10: CD38+ , HLA-DR+ (%) | CD8/Effector/PD1 (%) | CD8/Q4: CD45RO- , CD27- (%) |
| CD4/TEM/Q11: CD38+ , HLA-DR- (%) | CD8/Effector/PDL1 (%) | CD8/TNF (%) |
| CD4/TEM/Q12: CD38- , HLA-DR- (%) | CD8/Effector/TIGIT (%) |  |
| T cell/CD8 (%) | CD8/Effector/Tim3 (%) |  |
| T cell/CD8/Effector (%) | CD8/TIGIT (%) |  |
| T cell/CD8/Effector/CCR5+ (%) | CD8/Tim3 (%) |  |
| T cell/CD8/Effector/CD38+ (%) |  |  |
| T cell/CD8/Effector/CXCR5+ (%) |  |  |
| T cell/CD8/Effector/HLA-DR+ (%) |  |  |
| T cell/CD8/Effector/ICOS+ (%) |  |  |
| T cell/CD8/Effector/Ki67+ (%) |  |  |
| T cell/CD8/Effector/Q9: CD38- , HLA-DR+ (%) |  |  |
| T cell/CD8/Effector/Q10: CD38+ , HLA-DR+ (%) |  |  |
| T cell/CD8/Effector/Q11: CD38+ , HLA-DR- (%) |  |  |
| T cell/CD8/Effector/Q12: CD38- , HLA-DR- (%) |  |  |
| T cell/CD8/CCR5+ (%) |  |  |
| T cell/CD8/CD38+ (%) |  |  |
| T cell/CD8/CXCR5+ (%) |  |  |
| T cell/CD8/HLA-DR+ (%) |  |  |
| T cell/CD8/ICOS+ (%) |  |  |
| T cell/CD8/Ki67+ (%) |  |  |
| T cell/CD8/Naive (%) |  |  |
| T cell/CD8/Naive/CCR5+ (%) |  |  |
| T cell/CD8/Naive/CD38+ (%) |  |  |
| T cell/CD8/Naive/CXCR5+ (%) |  |  |
| T cell/CD8/Naive/HLA-DR+ (%) |  |  |
| T cell/CD8/Naive/ICOS+ (%) |  |  |
| T cell/CD8/Naive/Ki67+ (%) |  |  |
| T cell/CD8/Naive/Q9: CD38- , HLA-DR+ (%) |  |  |
| T cell/CD8/Naive/Q10: CD38+ , HLA-DR+ (%) |  |  |
| T cell/CD8/Naive/Q11: CD38+ , HLA-DR- (%) |  |  |
| T cell/CD8/Naive/Q12: CD38- , HLA-DR- (%) |  |  |
| T cell/CD8/Q2: CD45RO+ , CD27+ (%) |  |  |
| T cell/CD8/Q2: CD45RO+ , CD27+/TCM \| Freq. of CD8 (%) |  |  |
| T cell/CD8/Q2: CD45RO+ , CD27+/TCM/CCR5+ (%) |  |  |
| T cell/CD8/Q2: CD45RO+ , CD27+/TCM/CD38+ (%) |  |  |
| T cell/CD8/Q2: CD45RO+ , CD27+/TCM/CXCR5+ (%) |  |  |
| T cell/CD8/Q2: CD45RO+ , CD27+/TCM/HLA-DR+ (%) |  |  |
| T cell/CD8/Q2: CD45RO+ , CD27+/TCM/ICOS+ (%) |  |  |
| T cell/CD8/Q2: CD45RO+ , CD27+/TCM/Ki67+ (%) |  |  |
| T cell/CD8/Q2: CD45RO+ , CD27+/TCM/Q9: CD38- , HLA-DR+ (%) |  |  |
| T cell/CD8/Q2: CD45RO+ , CD27+/TCM/Q10: CD38+ , HLA-DR+ (%) |  |  |
| T cell/CD8/Q2: CD45RO+ , CD27+/TCM/Q11: CD38+ , HLA-DR- (%) |  |  |
| T cell/CD8/Q2: CD45RO+ , CD27+/TCM/Q12: CD38- , HLA-DR- (%) |  |  |
| T cell/CD8/Q2: CD45RO+ , CD27+/TTM \| Freq. of CD8 (%) |  |  |
| T cell/CD8/Q2: CD45RO+ , CD27+/TTM/CCR5+ (%) |  |  |
| T cell/CD8/Q2: CD45RO+ , CD27+/TTM/CD38+ (%) |  |  |
| T cell/CD8/Q2: CD45RO+ , CD27+/TTM/CXCR5+ (%) |  |  |
| T cell/CD8/Q2: CD45RO+ , CD27+/TTM/HLA-DR+ (%) |  |  |
| T cell/CD8/Q2: CD45RO+ , CD27+/TTM/ICOS+ (%) |  |  |
| T cell/CD8/Q2: CD45RO+ , CD27+/TTM/Ki67+ (%) |  |  |
| T cell/CD8/Q2: CD45RO+ , CD27+/TTM/Q9: CD38- , HLA-DR+ (%) |  |  |
| T cell/CD8/Q2: CD45RO+ , CD27+/TTM/Q10: CD38+ , HLA-DR+ (%) |  |  |
| T cell/CD8/Q2: CD45RO+ , CD27+/TTM/Q11: CD38+ , HLA-DR- (%) |  |  |
| T cell/CD8/Q2: CD45RO+ , CD27+/TTM/Q12: CD38- , HLA-DR- (%) |  |  |
| T cell/CD8/Q9: CD38- , HLA-DR+ (%) |  |  |
| T cell/CD8/Q10: CD38+ , HLA-DR+ (%) |  |  |
| T cell/CD8/Q11: CD38+ , HLA-DR- (%) |  |  |
| T cell/CD8/Q12: CD38- , HLA-DR- (%) |  |  |
| T cell/CD8/TEM (%) |  |  |
| T cell/CD8/TEM/CCR5+ (%) |  |  |
| T cell/CD8/TEM/CD38+ (%) |  |  |
| T cell/CD8/TEM/CXCR5+ (%) |  |  |
| T cell/CD8/TEM/HLA-DR+ (%) |  |  |
| T cell/CD8/TEM/ICOS+ (%) |  |  |
| T cell/CD8/TEM/Ki67+ (%) |  |  |
| T cell/CD8/TEM/Q9: CD38- , HLA-DR+ (%) |  |  |
| T cell/CD8/TEM/Q10: CD38+ , HLA-DR+ (%) |  |  |
| T cell/CD8/TEM/Q11: CD38+ , HLA-DR- (%) |  |  |
| T cell/CD8/TEM/Q12: CD38- , HLA-DR- (%) |  |  |
