# Supplementary material for: T cell immune discriminants of HIV reservoir size in a pediatric cohort of perinatally infected individuals
Source: PLoS Pathog. 2021 Apr 26;17(4):e1009533. doi: 10.1371/journal.ppat.1009533 (PMC8112655; doi:10.1371/journal.ppat.1009533)
Supplement: S2 Table — (DOCX) [file ppat.1009533.s002.docx]

**Supplementary Table 2:**

|  | **Overall** | **Cluster 1** | **Cluster 2** | **Cluster 3** | **p-value** |
| --- | --- | --- | --- | --- | --- |
|  | ***N=34*** | ***N=9*** | ***N=18*** | ***N=7*** |  |
| Time to suppression (months) | 4.26 [2.50;5.43] | 3.67 [2.49;5.51] | 4.59 [3.31;9.17] | 4.13 [1.76;4.77] | 0.565 |
| Rebound (ever): |  |  |  |  | 0.792 |
| No | 30 (88.2%) | 8 (88.9%) | 15 (83.3%) | 7 (100%) |  |
| Yes | 4 (11.8%) | 1 (11.1%) | 3 (16.7%) | 0 (0.00%) |  |
| Spike (ever): |  |  |  |  | 0.571 |
| No | 29 (85.3%) | 8 (88.9%) | 16 (88.9%) | 5 (71.4%) |  |
| Yes | 5 (14.7%) | 1 (11.1%) | 2 (11.1%) | 2 (28.6%) |  |
| Virological failure (ever): |  |  |  |  | 0.711 |
| No | 32 (94.1%) | 9 (100%) | 16 (88.9%) | 7 (100%) |  |
| Yes | 2 (5.88%) | 0 (0.00%) | 2 (11.1%) | 0 (0.00%) |  |
