# Supplementary material for: T cell immune discriminants of HIV reservoir size in a pediatric cohort of perinatally infected individuals
Source: PLoS Pathog. 2021 Apr 26;17(4):e1009533. doi: 10.1371/journal.ppat.1009533 (PMC8112655; doi:10.1371/journal.ppat.1009533)
Supplement: S3 Table — (DOCX) [file ppat.1009533.s003.docx]

| List of 96 genes included for the fluidigm experiments | | | | | | | | | | | |
| --- | --- | --- | --- | --- | --- | --- | --- | --- | --- | --- | --- |
| **ACTB** | **APOBEC3G** | **BATF** | **BCL2** | **BCL2A1** | **BCL6** | **BIRC5** | **BST2** | **BTLA** | **CASP3** | **CASP8** | **CCR2** |
| **CCR5** | **CCR6** | **CCR7** | **CD160** | **CD244** | **CD38** | **CD40L** | **CD69** | **CD74** | **CDK9** | **CDKN2A** | **CTLA4** |
| **CXCL10** | **CXCR3** | **CXCR5** | **DUSP4** | **DUSP6** | **EEFIA1** | **EGR2** | **EOMES** | **FAS** | **FOXP3** | **GAPDH** | **GATA3** |
| **GMCSF** | **GZMB** | **HAVCR2** | **ICOS** | **ID2** | **IFI16** | **IFNAR2** | **IFNG** | **IL10RA** | **IL12RB2** | **IL15RA** | **IL1B** |
| **IL2** | **IL21** | **IL21R** | **IL27** | **IL2RA** | **IL4** | **IL6RA** | **IL6ST** | **IL7R** | **IRF4** | **KLRG1** | **KRAS** |
| **LAG3** | **MAF** | **MAPK3** | **MIP1A** | **MPEG1** | **MYC** | **NFATC1** | **NFKB1** | **PDCD1** | **POU2AF1** | **PRDM1** | **PRF1** |
| **PTEN** | **RCAN2** | **RORC** | **RUNX3** | **SAMHD1** | **SH2D1A** | **SIPR1** | **SIRT1** | **SLC2A1** | **STAT1** | **STAT3** | **STAT4** |
| **STAT5A** | **TBX21** | **TCF7** | **TGFB1** | **TIGIT** | **TNF** | **TNFRSF4** | **TNFRSF8** | **TNFRSF9** | **TNFSF10** | **TRIM5** | **XCL1** |

**Supplementary Table 3:**
