# Supplementary figures and images for: T cell immune discriminants of HIV reservoir size in a pediatric cohort of perinatally infected individuals
Source: PLoS Pathog. 2021 Apr 26;17(4):e1009533. doi: 10.1371/journal.ppat.1009533 (PMC8112655; doi:10.1371/journal.ppat.1009533)

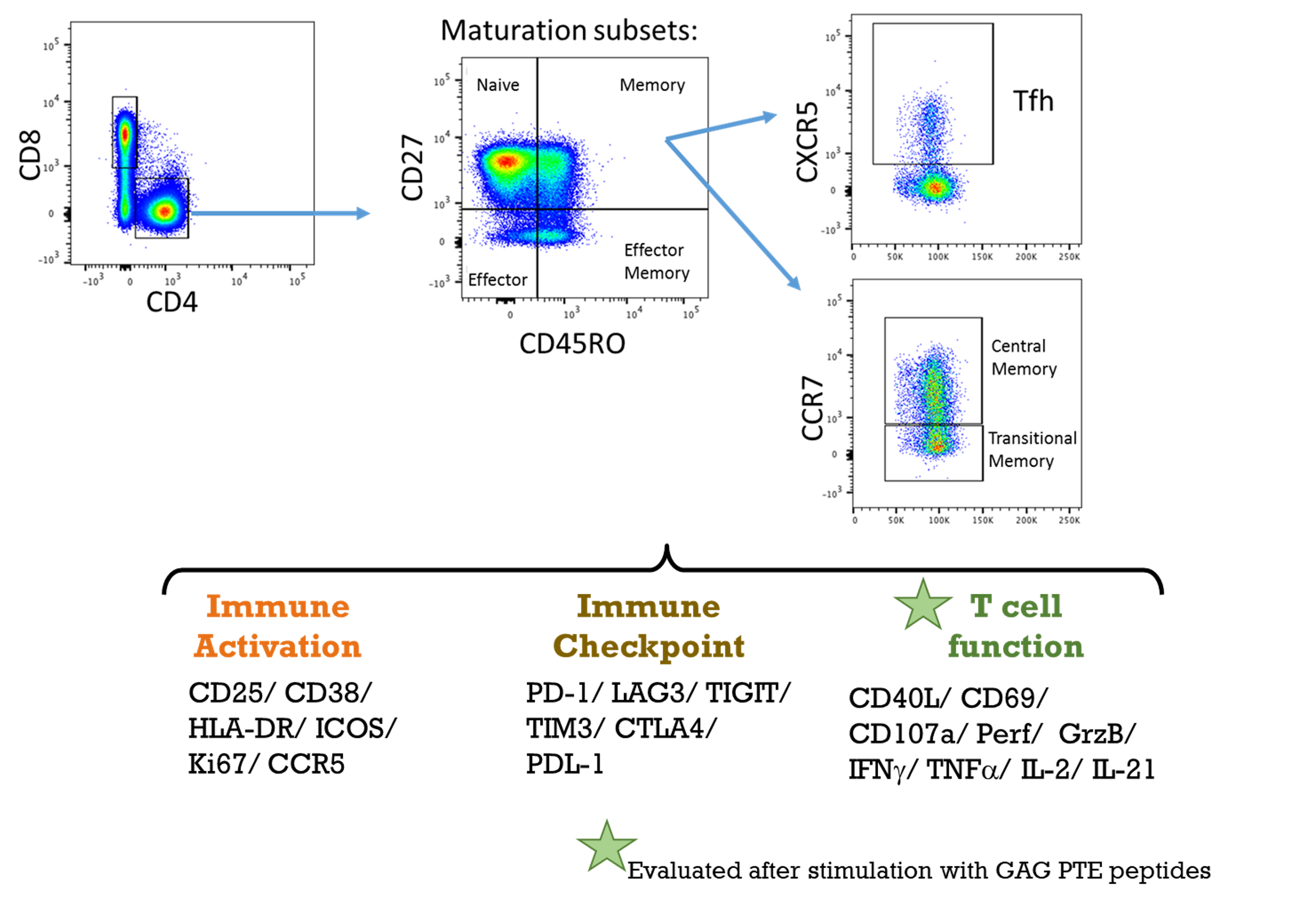

Supplement: S1 Fig — A) Example of gating strategy used for identification of the different CD4 and CD8 Maturational subsets. (TIF) [file ppat.1009533.s004.tif]

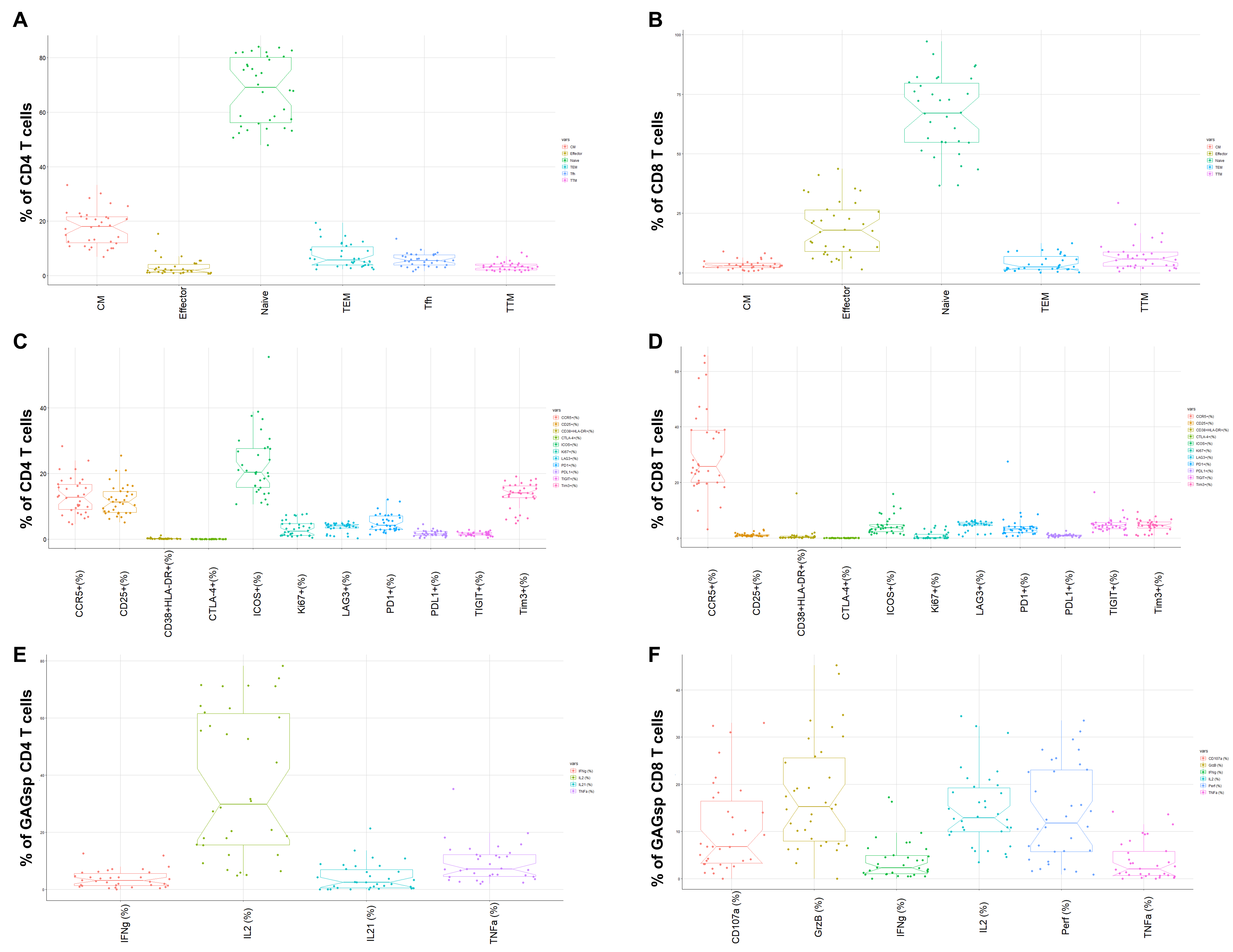

Supplement: S2 Fig — A-B) Distribution of the maturational subsets in CD4 (A) and CD8 (B) T cells. C-D) Distribution of the immune activation and immune checkpoint markers expression in total CD4 (C) and CD8 (D) T cells. E-F) Distribution of cytokines’ expression in HIV specific (CD40L+) CD4 (E) and HIV specific (CD69+) CD8 (F) T cells after stimulation with HIV GAG PTE peptides. (TIF) [file ppat.1009533.s005.tif]

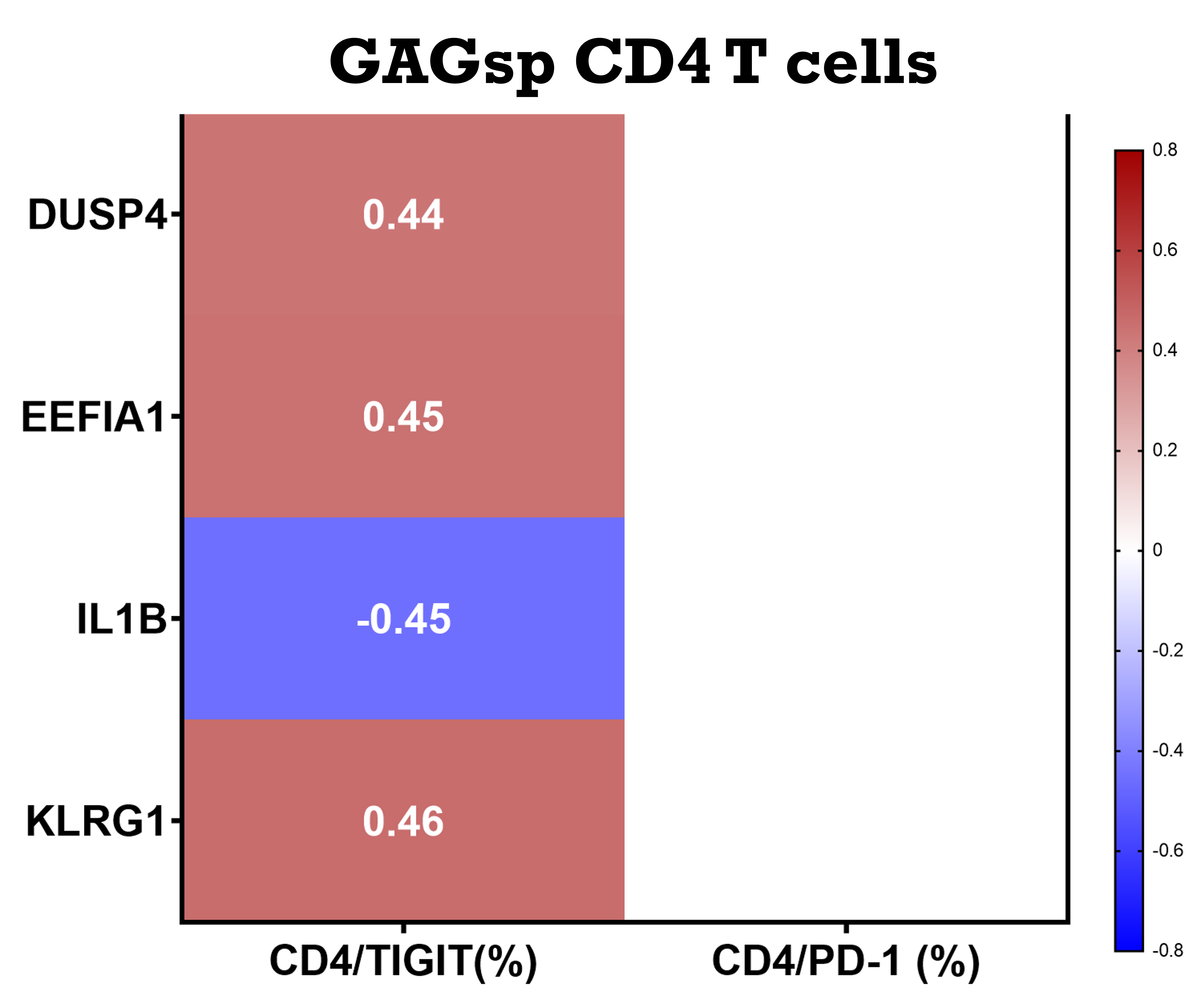

Supplement: S3 Fig — Non-significant correlations are reported in white. R value for every significant correlation is color coded with negative r value reported in blue while positive are reported in red. The darker the color the stronger the correlation. The actual R value is also reported inside every squares only for the significant correlations. (TIF) [file ppat.1009533.s006.tif]

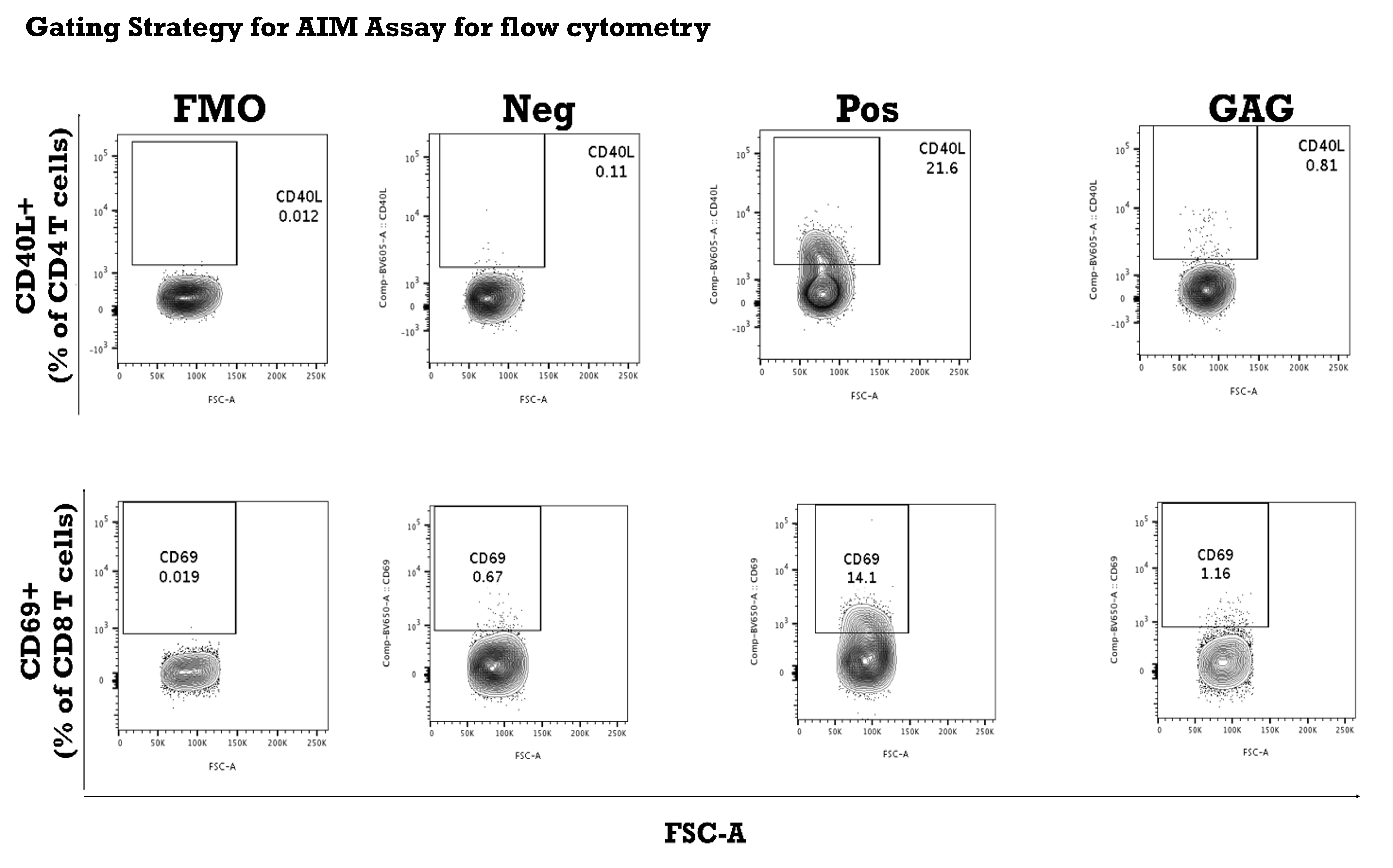

Supplement: S4 Fig — Gating control (FMO) was set to define the negative population. An unstimulated condition (Neg) and a stimulation using polyclonal stimulus SEB as positive control (Pos) were used to identify the cells responding to the stimulation. (TIF) [file ppat.1009533.s007.tif]

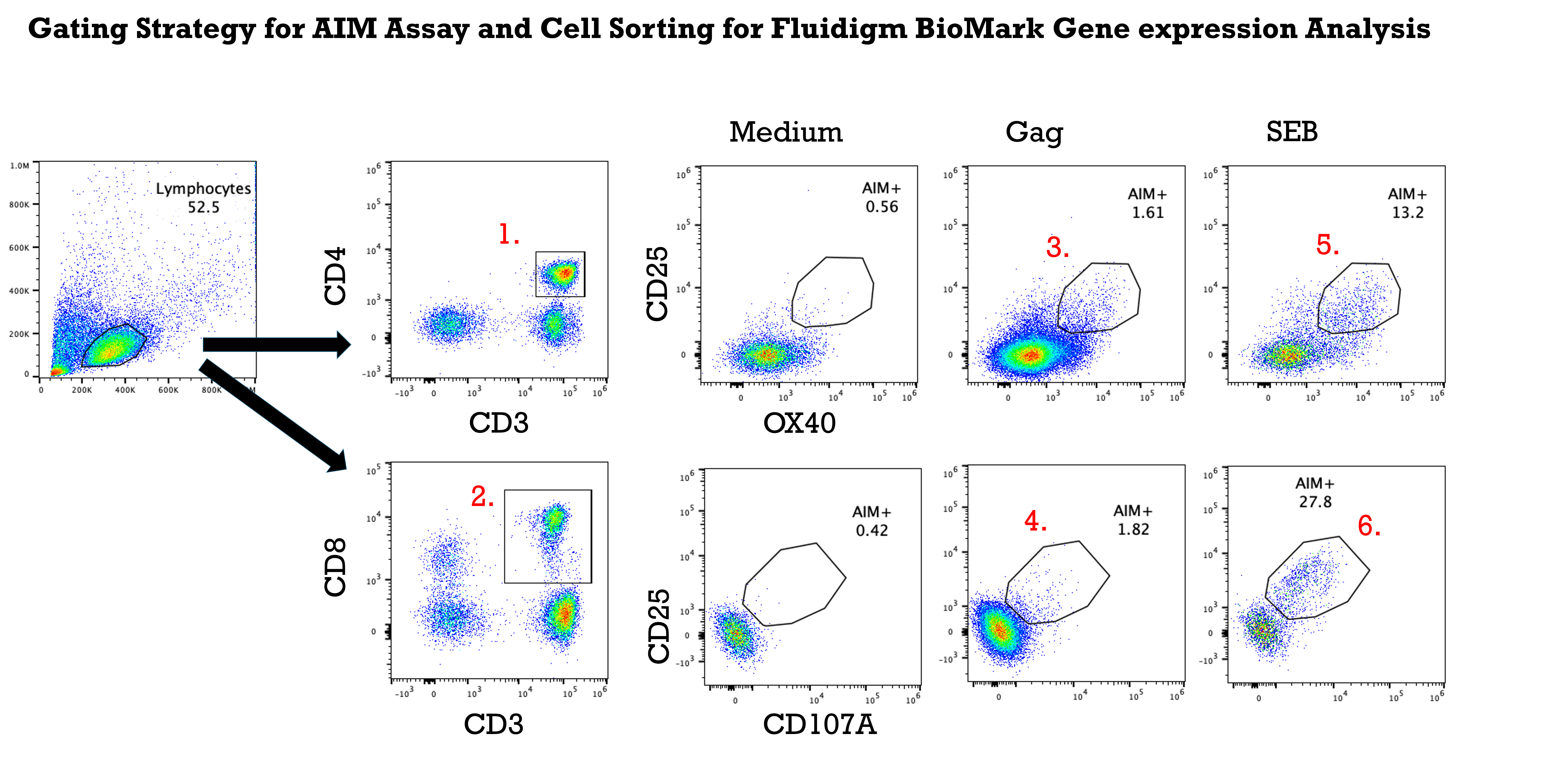

Supplement: S5 Fig — Numbered populations were sorted: 1 and 2 were from the medium (unstimulated) condition, 3 and 4 from the Gag-stimulated condition, and 5 and 6 from the SEB stimulated condition. (TIF) [file ppat.1009533.s008.tif]
